# ZW4864‐mediated inhibition of the β‐catenin/BCL9/BCL9L complex reveals therapeutic potential in bladder cancer

**DOI:** 10.1002/1878-0261.70292

**Published:** 2026-06-18

**Authors:** Roland Kotolloshi, Mandy Berndt‐Paetz, Eileen Lerner, Gregoire Najjar, Anca Azoitei, Krishna Pal Singh, Shailendra Kumar Gupta, Olaf Wolkenhauer, Cagatay Günes, Otmar Huber, Marc‐Oliver Grimm, Daniel Steinbach

**Affiliations:** ^1^ Department of Urology Jena University Hospital Germany; ^2^ Department of Urology University Medical Center Rostock Germany; ^3^ UroFors Consortium (Natural Scientists in Urological Research), German Society of Urology Düsseldorf Germany; ^4^ Department of Urology Leipzig University Germany; ^5^ Department of Urology Ulm University Hospital Germany; ^6^ Department of Systems Biology & Bioinformatics, Institute of Computer Science University of Rostock Germany; ^7^ Clinic and Policlinic for Dermatology, Venereology and Allergology University Medical Center Rostock Germany; ^8^ German Study Group of Bladder Cancer (DFBK e.V.), Munich Germany; ^9^ Department of Biochemistry II Jena University Hospital Germany

**Keywords:** BCL9, BCL9L, bladder cancer, *ex vivo* porcine urinary model, invasion, multicellular bladder cancer organoids, Wnt/β‐catenin signaling

## Abstract

Bladder cancer is the most prevalent malignancy of the urinary tract; nevertheless, its progression and treatment continue to pose substantial challenges. While the molecular mechanisms underlying bladder cancer progression remain unknown, the Wnt/β‐catenin signaling pathway has established roles in cancer progression. In this study, we investigated the contribution of BCL9(L) (coactivators of β‐catenin) to bladder cancer progression and evaluated the therapeutic potential of disrupting the β‐catenin/BCL9(L) complex. We demonstrate that BCL9L exerts oncogenic effects on the proliferation and invasion of bladder cancer cells and the knockdown of BCL9 also reduced the proliferation, migration, and invasion of bladder cancer cells, suggesting similar oncogenic roles. Pharmacological inhibition of the β‐catenin/BCL9(L) complex using ZW4864 suppresses a subset of Wnt/β‐catenin targets in bladder cancer cells, indicating the inhibition of canonical Wnt signaling. We show ZW4864 has an inhibitory effect on the proliferation, migration, and invasion of bladder cancer cells and impedes the growth and formation of multicellular bladder cancer organoids. These findings demonstrate the crucial role of BCL9 and BCL9L in bladder cancer progression and highlight the β‐catenin/BCL9(L) axis as a promising therapeutic target.

## Graphical abstract

BCL9 and BCL9L drive bladder cancer progression by enhancing β‐catenin signaling, promoting proliferation, migration, invasion, and organoid growth. Genetic depletion of BCL9(L) suppresses malignant phenotypes, while pharmacological disruption of the β‐catenin/BCL9(L) complex with ZW4864 inhibits canonical Wnt signaling and tumor‐associated cellular functions, identifying the β‐catenin/BCL9(L) axis as a promising therapeutic target in bladder cancer.

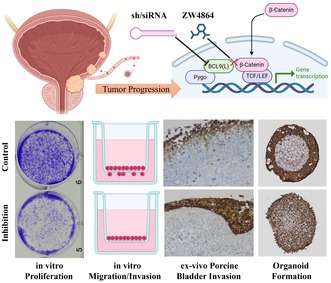

**Abbreviations**
BC: Bladder Cancer
DS2022: (Biovia) Discovery Studio 2022
EMT: Epithelial‐Mesenchymal Transition
EV: Enfortumab Vedotin
hBFs: Human Bladder Fibroblasts
hBSMCs: Human Bladder Smooth Muscle Cells
HEK293T: Human Embryonic Kidney 293 cells
MIBC: Muscle‐Invasive Bladder Cancer
NMIBC: Non‐Muscle‐Invasive Bladder Cancer
PCR: Polymerase Chain Reaction
PDX: Patient‐Derived Xenograft
qRT‐PCR: Quantitative Reverse Transcription PCR
STR: Short Tandem Repeat
UTR: Untranslated Region (5′‐ and 3′‐UTRs)

## 1 Introduction

Bladder cancer (BC) is the most prevalent malignancy in the urinary system and is among the most widespread cancers on a global scale. According to data from 2022, BC ranks as the ninth most prevalent cancer worldwide, with 613.791 new cases and 220.349 deaths. The incidence of the condition is approximately three times higher in men than in women, with 471.072 and 142.719 cases recorded, respectively [1]. This may be attributed to the primary risk factors associated with BC. Cigarette smoking has been identified as the most significant contributing factor to the development of BC, accounting for 50% of all diagnosed cases [2, 3]. Furthermore, occupational exposure to carcinogens in industrial areas has been identified as an additional risk factor for BC [4]. Bladder cancer originates from the urothelial layer of the bladder. Patients are initially diagnosed with either non‐muscle‐invasive bladder cancer (NMIBC) or muscle‐invasive bladder cancer (MIBC) in 80% and 20% of cases, respectively [5, 6]. The probability of progression after five years is up to 45%, which is considered to be of clinical importance [5, 7, 8]. The progression of bladder cancer is associated with a poor overall prognosis and survival rate. The disease often leads to metastasis and death [9, 10]. Early radical cystectomy, either as a stand‐alone treatment or in conjunction with adjuvant or neoadjuvant cisplatin‐based chemotherapy, or immune checkpoint therapy, remains the standard therapy for patients with high‐risk NMIBC and MIBC [11, 12, 13, 14, 15]. Locally advanced and metastatic bladder cancer patients may benefit from treatment with antibody‐drug conjugates such as enfortumab vedotin (EV) in the future [16]. However, these therapeutic options are associated with severe implications for patients and an overall negative impact on quality of life. Despite these intensive treatments, recurrence occurs in more than 40% of patients within three years [9, 17, 18]. Recently, the clinical evaluation of bladder‐preserving therapy for subgroups of high‐risk NMIBC patients and MIBC patients has come into focus. Trimodal therapies and local target‐specific treatments, such as intravesical FGFR3 inhibitor delivery systems, are currently being investigated in clinical trials [19]. However, for numerous molecular subtypes of BC, there is an absence of available targeted treatment. Consequently, there is an urgent need to identify novel therapeutic targets that will expand the portfolio of targets for the development of personalized treatment options.

The Wnt/β‐catenin pathway, also referred to as the canonical Wnt pathway, is a highly versatile pathway that plays a critical role in various cellular processes, including proliferation, apoptosis, migration, and invasion. It has been shown to be essential for a broad spectrum of biological processes, ranging from embryonic development to tissue homeostasis [20, 21, 22, 23]. As one of the highly conserved signaling pathways, dysfunction of Wnt/β‐catenin‐signaling leads to many diseases and several cancer types [24, 25, 26]. The role of Wnt/β‐catenin signaling in various tumor entities has been a subject of extensive analysis over many years, and its significance in bladder cancer is now emerging. A correlation between Wnt/β‐catenin signaling and BC progression stage, invasiveness, and poor prognosis has been demonstrated in several recent reports. Additionally, the maintenance and regulation of urothelial cancer stem cells was suggested to be attributable to Wnt signaling [27, 28, 29, 30]. Recently, additional targets have been identified that play a role in the growth, invasion, and metastasis of bladder cancer. These targets contribute to the regulation of Wnt/β‐catenin signaling [31, 32, 33, 34]. The β‐catenin protein plays a central role in the Wnt/β‐catenin signaling. It is normally continuously degraded under strict regulation. Upon activation, the β‐catenin protein accumulates in the cytoplasm and nucleus, forming a functional complex with the TCF/LEF transcription factor, including PYGO, coactivators B‐cell CLL/lymphoma 9/L (BCL9/L) and other cofactors to promote Wnt signaling [26, 35, 36, 37]. In previous studies, we demonstrated the effect of Wnt/β‐catenin signaling on BC cell lines in response to Wnt pathway inhibition and BCL9L depletion, suggesting a role of this pathway in BC progression [38]. BCL9 and BCL9L are homologous genes that share 34.8% amino acid sequence identity and three conserved homology domains: HD1, HD2, and HD3 [39]. Through the HD2 domain, the BCL9 and BCL9L proteins interact with β‐catenin, thereby activating Wnt/β‐catenin signaling [40, 41, 42]. Both BCL9 and BCL9L are associated with chromosomal abnormalities and are frequently overexpressed. They are also crucial drivers of several tumor entities, including colorectal cancer, hepatocellular carcinoma, myeloma, and breast cancer. Furthermore, knockdown experiments of BCL9 and BCL9L promoted an antitumor response and increased survival in animal xenograft models [43, 44, 45, 46, 47, 48, 49]. Currently, BCL9 is studied more intensively in cancer than its homolog, BCL9L. Independent studies have suggested that the molecular mechanisms of both BCL9 and BCL9L in cancer cell invasion and disease progression are mediated by further activation of Wnt/β‐catenin signaling [43, 44, 45, 50, 51, 52, 53, 54, 55, 56].

In our previous work, we identified several genes involved in the Wnt/β‐catenin signaling and other pathways that were mutated in the coding sequences as well as in the 5′‐ and 3′‐untranslated regions (UTRs) of tumors from BC patients with progressive disease [57]. The BCL9L UTR exhibited mutations in three out of 11 patients. Our immunohistochemical analysis revealed that the BCL9L protein level was predominantly higher in MIBC than in NMIBC, suggesting an association between BCL9L and tumor stage. To the best of our knowledge, this was the first study to demonstrate that the knockdown of BCL9L represses the proliferation, migration, and invasion of BC cells, suggesting an oncogenic role of BCL9L in BC [38]. However, the role of BCL9, the homolog of BCL9L, remains to be elucidated in BC. Therefore, the objectives of this study were to analyze the functional role of BCL9 in BC cell lines and determine whether treatment with the β‐catenin/BCL9 inhibitor ZW4864 elicits an antitumor response in BC.

## 2 Materials and methods

### 2.1 Cell culture

The human bladder cancer cell lines Cal29 (RRID:CVCL_1808; supplier: DSMZ GmbH Cat. No. ACC 515, year 2010) and J82 (RRID:CVCL_0359; supplier: Elabscience, Cat.No. EP‐CL‐0125, year 2021) were cultured in DMEM with GlutaMAX (Thermo Scientific, Germany) supplemented with 10% (v/v) fetal bovine serum (FBS), penicillin (100 U/mL), and streptomycin (100 μg/mL). The short tandem repeat (STR) profile of the cells was analyzed using the PowerPlex 16 System (Promega, Germany) and validated through online STR analysis provided by the German Collection of Microorganisms and Cell Cultures (DSMZ). Primary human bladder fibroblasts (hBFs, FC‐0050) and human bladder smooth muscle cells (hBSMCs, FC‐0043) were both purchased from CellSystems (Troisdorf, Germany). The hBFs were cultured in FibroLife^®^ S2 medium (LL‐0011), and the hBSMCs were cultured in VascuLife^®^ SMC medium (LL‐0014; Lifeline Cell Technology, Frederick, MD, USA). The accutase cell detachment solution (PanBiotech, Aidenbach, Germany) solution was applied to obtain single‐cell suspensions. All cells were grown in suitable media and maintained in a humidified atmosphere at 37 °C with 5% CO_2_. Cell cultures were periodically tested for mycoplasmas using PCR test (PromoKine, PK‐CA91‐1024).

### 2.2 Transient BCL9 knockdown by siRNA transfection

In the transient knockdown experiments, Cal29 and J82 cells were seeded, and the following day, the cells were transfected with 60 nM of a pooled siRNA targeting BCL9 (SI03104759, SI02632938, SI00062734, and SI00062720, Qiagen, Germany) and with 60 nm siControl (Allstars negative control siRNA, SI0365031, Qiagen, Germany) using INTERFERin reagent, following the manufacturer's protocol (Polyplus, USA). Briefly, 60 nm siRNA was diluted in 200 μL Opti‐MEM I reduced serum medium (31 985 062, Thermo Scientific, Germany) and 16 μL of INTERFERin reagent. The transfection mixture was mixed by vortexing, incubated for 10 min at room temperature, and added dropwise onto the cells in serum‐containing medium. The plate was then gently shaken and subsequently returned to the 37 °C incubator. The following day, the medium was replaced with growth medium.

### 2.3 Generation of stable BCL9L knockdown Cal29 cells using a lentiviral system

The lentiviral psi‐LVRU6GP plasmid system was utilized to generate stable BCL9L knockdown. Three different shRNA target sequences against BCL9L (HSH007858‐a‐LVRU6GP, HSH007858‐d‐LVRU6GP, HSH007858‐e‐LVRU6GP, GeneCopoeia, MD, USA) and one shControl (CSHCTR001‐LVRU6GP, GeneCopoeia, MD, USA) were utilized. All vectors contain an EGFP expression and puromycin resistance cassette. Prior to transfection, 1.5 × 10^6^ HEK293T cells were seeded in a 6 cm culture dish, and after 24 h, the cells reached 80–90% confluency. Subsequently, the cells were cotransfected with the psi‐LVRU6GP vector and three packaging pMDL, pRSV and pVSV‐g plasmids (kindly provided by the Department of Gynecology and Reproductive Medicine, Jena University Hospital). The Lipofectamine 2000 reagent (Thermo Scientific, MA, USA) was used to produce viral particles. Cal29 cells were infected with the viral particles and continuously selected with 2 μg/mL puromycin in the medium. The knockdown efficiency was evaluated following selection by qRT‐PCR and western blotting. However, the knockdown was successful for only one clone compared with the shControl (HSH007858‐a‐LVRU6GP positive clone), which was used for further functional analysis.

### 2.4 Assessment of cell proliferation by crystal violet staining

Crystal violet staining was utilized as an indirect method for analyzing cell proliferation. For these assays, 20 × 10^4^ Cal29 or 4 × 10^4^ J82 cells were seeded on 6‐well cell culture plates. After 24 h of culture, the cells were either transfected with siRNA or treated with the inhibitor ZW4864. To achieve transient knockdown of BCL9, Cal29 and J82 cells were transfected with 60 nm pooled of siBCL9, including a siRNA negative control, as described previously. In order to inhibit the Wnt/β‐catenin/BCL9(L) signaling axis, the cells were treated with 10 μm, 20 μm or 40 μm ZW4864, or with 0.1% (v/v) DMSO used as a control. After 6 days, the cells were washed once in PBS buffer, fixed with 2% (v/v) glutaraldehyde solution in PBS for 10 min and stained with 0.1% (w/v) crystal violet solution for 30 min. For the purpose of analysis, the stained cells were solubilized with lysis solution (0.1 m sodium citrate, 50% (v/v) ethanol, pH 4.2) for 30 min. Thereafter, the absorbance was measured on an Infinite M200 Pro reader at a wavelength of 590 nm (Tecan, Austria). At least three independent biological replicates were performed, including two technical replicates for each experiment.

### 2.5 Apoptosis analysis by flow cytometry

For the analysis of apoptosis and cell death, 2 × 10^5^ J82 or 2.5 × 10^4^ Cal29 cells were seeded on 6 cm cell culture dishes. The following day, the cells were transfected with 60 nm pooled siBCL9 or treated with 10 μm, 20 μm or 40 μm ZW4864 (MedChemExpress LLC, NJ, USA). After 2 days, the transfected or treated cells were evaluated using a BD Accuri C6 Plus Flow Cytometry system (BD Pharmingen, Germany) in conjunction with a FITC Annexin V apoptosis detection kit (556 547, BD Pharmingen, Germany) according to the manufacturer's protocol. The BD Accuri C6 Plus software was utilized to acquire and analyze the data using specific templates for annexin V‐FITC (FL1 channel, 530 nm filter) and PI (FL2 channel, 575 nm filter). At least 1 × 10^4^ events were used for each measurement, excluding cell debris, by setting appropriate light scatter gates. At least three independent biological replicates were included.

### 2.6 Real‐time migration and invasion analysis using the xCELLigence RTCA system

The real‐time migration and invasion assays were performed with the xCELLigence RTCA System (ACEA Bioscience, CA, USA) according to the manufacturer's protocol. For transient BCL9 knockdown, Cal29 or J82 cells were transfected with 60 nm of a pooled siBCL9 as previously described and cultured for three days. To inhibit the β‐catenin/BCL9(L) complex, the cells were treated with 20 μM ZW4864, including a DMSO control, for 2 days. Following the manufacturer's protocol for the cell invasion and migration assays, the transfected or treated cells were trypsinized, and the cell pellet was resuspended in serum‐free medium (SFM). To the upper chamber of a special CIM plate, 2 × 10^4^ Cal29 or 2.5 × 10^4^ J82 cells in 100 μL FSM were added for migration analysis. For the invasion assay, 4 × 10^4^ Cal29 or 5 × 10^4^ J82 cells in 100 μL SFM were seeded in the upper chamber, which was precoated with 1:40 diluted Matrigel (Corning, New York, USA). For both migration and invasion experiments, the lower chamber of the trans‐well contained medium supplemented with 10% (v/v) FCS as a chemoattractant. The acquisition of data was conducted continuously, with the resulting data expressed as cell index, a metric that reflects migration and invasion events. Three independent biological replicates were performed, including three technical replicates for each experiment.

### 2.7 Boyden chamber invasion assay

Prior to the seeding process in the Boyden chamber, Cal29 control cells (shControl) or cells with stable BCL9L knockdown (shBCL9L) were starved in serum‐free media for 48 h. Thereafter, 1 × 10^4^ cells were seeded in serum‐free medium on the Matrigel^®^ layer in the upper chamber of the setup. The inserts were subsequently placed in a 12‐well plate containing 1 mL of complete DMEM and incubated for 48 h. Invasive cells located on the bottom side of the insert were fixed and then stained with crystal violet. Pictures from five areas were obtained using a Zeiss Axio.Imager M2 Microscope under a 10x objective lens, and the number of invasive cells was counted. Statistical analysis was performed using Student's *t*‐test to compare the two groups.

### 2.8 *Ex vivo* porcine urinary bladder model

The *ex vivo* porcine urinary bladder assay was performed as described by Wezel et al. (2021) [58]. Briefly, porcine bladders were obtained from a local abattoir, subsequently opened, and thoroughly washed three times in DPBS containing 3% (v/v) penicillin/streptomycin. The samples were then incubated overnight at 4°C in 0.5% (w/v) Dispase^®^ II (Cat No. D4693, Sigma–Aldrich, St. Louis, MO, USA) to loosen the urothelial cells. The following day, the epithelial layer was scraped, and 1 cm^2^ bladder pieces were cut and placed in 70 μm cell strainer inserts in a six‐well plate containing 5 mL of optimized Waymouth medium. The medium was supplemented with 10% (v/v) FBS, 300 μg/mL L‐ascorbic acid, 0.45 μg/mL ferrous sulfate heptahydrate, and 2 μg/mL hydrocortisone (Sigma–Aldrich, St. Louis, MO, USA) and maintained in culture for 24 h. The following day, 1 × 10^6^ Cal29‐shControl or Cal29‐shBCL9L cells were pelleted, resuspended in 20 μL of complete DMEM, and then seeded onto the de‐epithelialized bladder inside a ring. The air–liquid interface was maintained with 5 mL of medium, which was changed every other day. After 14 days, the tissue was harvested, fixed in 10% (v/v) formalin, and embedded in paraffin. Next, the paraffin blocks were sectioned into 4‐μm thick slices and then stained with hematoxylin and eosin or using an anti‐HLA Class 1 antibody to identify colonized human cytokeratin positive urothelial cells (Cat.No. ab70328, abcam).

### 2.9 Spheroid formation of bladder cancer cells and treatment

For spheroid volume analysis, 80% confluent J82 cells were trypsinized and collected by centrifugation (100 × g for 10 min), followed by resuspension with fresh medium. Subsequently, 1 × 10^3^ J82 cells were seeded in a U‐bottom cell repellent surface 96‐well microplate (650 970, Greiner Bio‐One, Germany) and centrifuged at 100 × g for 3 min to form 3D spheroid structures. After 24 h, J82 spheroids were treated with 10 μm, 20 μm or 40 μm ZW4864, including a DMSO control, for 30 days. Day 0 referred to the beginning of the treatment with ZW4864, and images were taken every 3 days by a Primovert microscope with an Axiocam ERc 5 s camera (Zeiss, Jena, Germany). Data analysis for spheroid volume was conducted using the MATLAB R20019a and the AnaSP software. The experiments were conducted with a minimum of three independent biological replicates.

### 2.10 Generation of multicellular bladder cancer organoids

Multicellular bladder cancer organoids were generated according to the methodology described in the study by Berndt‐Paetz et al. 2023 [59]. In brief, the ultra‐low attachment microplate method was used to generate BC organoids. In this study, Cal29) or J82 cells were mixed with human bladder fibroblasts (hBF, FC‐0050, Cell Systems, Troisdorf, Germany) and human bladder smooth muscle cells (hBSMC, FC‐0043, Cell Systems, Troisdorf, Germany) at equal densities (5 × 10^3^ cells each). Subsequently, the cell suspensions (1.5 × 10^4^ cells per well) were centrifuged (250 × g, 5 min) in Nunclon™ Sphera™ 96‐well ULA round bottom plates (Thermo Fisher Scientific, Dreieich, Germany). Self‐organizing BC organoids were cultured in a 1:1:1 mixture of cell type‐specific media for 4 days, at which point histological analysis and DAB immunostaining were conducted for the purpose of morphological characterization. In order to assess the efficacy of β‐catenin/BCL9(L) inhibition, BC organoids were pre‐formed for 3 days following the addition of ZW4864. BC organoids were freshly prepared for each experiment and maintained in culture for a maximum period of 10 days.

### 2.11 Treatment responses in BC organoids

The β‐catenin/BCL9(L) inhibitor ZW4864 was added after 3 days of organoid culture. BC organoids were incubated with ZW4864 (10 μm, 20 μm, 40 μm or 0.1% (v/v) DMSO) diluted in culture medium. The ULA plates were placed into the IncuCyte^®^ Live‐Cell Analysis System (Sartorius, Epsom, UK) after the addition of ZW4864. Scanning was repeated every 4 h over a period of 10 days was scheduled for live‐cell imaging. Organoid viability was determined after 3, 7 and 10 days using the CellTiter‐Glo^®^ 3D Cell Viability Assay (Promega, Mannheim, Germany) measured on a SpectraMax M5 microplate reader (Molecular Devices, Sunnyvale, USA). The organoid morphology was analyzed by histological processing.

### 2.12 Organoid histology

Organoids were fixed with 4% (w/v) paraformaldehyde and embedded in HistoGel (Thermo Fisher Scientific, Dreieich, Germany). Serial sections with thickness of 4 μm were immunostained following paraffin embedding. For DAB staining, the sections were deparaffinized, followed by antigen retrieval using heat‐induced epitope retrieval buffer (pH 9.0; Zytomed Systems, Berlin, Germany) for 40 min at 100°C. Following the blocking of the endogenous peroxidase, the sections were incubated overnight at 4°C with the following primary antibodies: anti‐panCK (1:250; Cat. No.: C2931, Sigma‐Aldrich, Munich, Germany), anti‐vimentin (1:200; Cat. No.: V6389, Sigma‐Aldrich, Munich, Germany), anti‐alpha smooth muscle actin (1:1000; Cat. No.: A2547, Sigma‐Aldrich, Munich, Germany), and anti‐Ki67 (1:50; Cat. No. M0722, Dako, Glostrup, Denmark). The biotin‐linked goat‐anti‐mouse secondary antibody (1:400; Cat. No. BA‐9200.1.5; Vector Laboratories, Newark, NJ, USA) was incubated for 45 min at room temperature. Following a coupling step with horseradish peroxidase streptavidin, DAB was used for visualization, and the nuclei were counterstained with hematoxylin. The acquisition of images was facilitated by a Keyence BZ‐X800 microscope (Keyence Corporation, Osaka, Japan). An analysis of the DAB stains was conducted in Fiji, including ImageJ version 2.16.0 software.

### 2.13 Total RNA extraction

For the efficient extraction of total RNA from cell culture experiments Triazol reagent protocol (Thermo Scientific, MA, USA) was used. Triazol is based on the guanidine isothiocyanate/phenol method. Briefly, at the end of the experiments, the Cal29 and J82 cells were washed once with PBS and lysed with 1 mL Triazol, followed by phase separation as described in the Triazol manufactures protocol (Thermo Fisher Scientific, USA). After dissolving in DEPC water, the total RNA was quantified using Qubit 3.0 fluorometer (Invitrogen, Thermo Fischer Scientific, MA, USA) and analyzed using an Agilent 2200 TapeStation (Agilent, Waldbronn, Germany) according to the manufacturer's protocol. The RNA was stored at −80 °C.

### 2.14 cDNA synthesis and reverse transcription quantitative PCR

Two micrograms of total RNA were transcribed into cDNA using the GoScript Reverse Transcription System Kit (Promega, Mannheim, Germany) according to the manufacturer's protocol. The cDNA was diluted 1:8 with DEPC water and stored at −20 °C. mRNA expression analysis was performed using LightCycler 480 SYBR Green I master mix and a LightCycler 480 instrument (Roche Applied Science, Germany) utilizing specific primers for each target. The second derivative maximum method of the LightCycler software was used to extract the C_p_ value. For the purpose of mRNA expression analysis, the targets were normalized to the housekeeping genes RPS13 and RPS23. The Roche Diagnostic method was then employed for evaluation, with primer efficiency being taken into account [60, 61].

### 2.15 Protein analysis by western blotting

To extract total protein, the cells were collected and lysed in a 5 × volume of cold NETN buffer (100 mm NaCl, 20 mm Tris/HCl pH 8.0, 1 mm EDTA, 0.5% (v/v) NP‐40) supplemented with a protease inhibitor cocktail (Merck, Darmstadt, Germany) and incubated on ice for 10 min. Afterwards, the cells were then subjected to three cycles of freezing and thawing. The protein concentration was measured with a Coomassie (Bradford) protein assay kit (Thermo Scientific, Germany), and the samples were stored at −80 °C. For the western blotting, 30 μg of total protein was separated by 10% SDS/PAGE according to molecular size and transferred onto polyvinylidene fluoride (PVDF) membranes by the xCell II Blot Module (Invitrogen, USA). The membranes were then incubated for one hour with a 5% (w/v) fat‐free milk solution at room temperature. For the immunodetection, the primary antibodies were incubated overnight at 4 °C, while the horseradish peroxidase‐conjugated secondary antibodies were incubated for 1 h at room temperature. The membranes were incubated with chemiluminescence (ECL) reagent (GE Healthcare, Solingen, Germany) and the resulting light emission was detected with a GBox Chemi XX6 Imager (Syngene, Cambridge, GB). The following primary antibodies were utilized: anti‐BCL9L (HPA049370, Atlas Antibodies, Sweden, 1:1000 dilution), anti‐BCL9 (ab37305, Abcam, Germany, 1:1000 dilution) and anti‐GAPDH (sc‐47 724, Santa Cruz, Germany, 1:2500 dilution). The following secondary antibodies were used: anti‐mouse IgG‐HRP (sc‐516 102, Santa Cruz, Germany, 1:5000 dilution) and anti‐rabbit IgG‐HRP (sc‐2370, Santa Cruz, Germany, 1:5000 dilution).

### 2.16 Ligand preparation for computational and pharmacokinetic analyses

The chemical structure of ZW4864 was retrieved from the PubChem database (Compound CID: 156180088). Ligand preparation was performed using the Prepare Ligands tool in BIOVIA Discovery Studio 2022 (DS2022). This protocol ensured systematic preprocessing of the compound, including the generation of 3D structures, geometry optimization, and energy minimization. The purpose of these steps was to remove steric clashes and achieve conformational stability prior to docking analyses.

### 2.17 Off‐target profiling using the ligand profiler

Off‐target profiling of ZW4864 was conducted using the Ligand Profiler module in DS2022 (BIOVIA Discovery Studio 2022). This protocol maps a query compound to pharmacophore models stored in the PharmaDB database, which contains more than 250 000 pharmacophore models derived from 16 304 experimentally validated protein–ligand complexes in the 2017 release of the sc‐PDB database (https://drugdesign.unistra.fr/scPDB/).

ZW4864 was screened against the PharmaDB pharmacophore library to predict potential protein targets on the basis of pharmacophore–ligand complementarity. The resulting protein hits were filtered to retain only human models. A Fit‐Value score, which represents the degree of geometric and chemical alignment between ZW4864 and each pharmacophore model, was calculated for every hit. The top 10 targets with the highest fit value scores were selected for enrichment and pathway analysis.

### 2.18 Molecular docking of ZW4864 with β‐catenin

The interaction between ZW4864 and β‐catenin was evaluated using the flexible CDOCKER protocol implemented in DS2022 [62]. The crystal structure of β‐catenin (PDB ID: 2GL7) was obtained from the Protein Data Bank (PDB) and prepared using the Prepare Protein protocol in DS2022. This step corrected missing atoms in incomplete residues, built missing loop regions, resolved alternate conformations, and optimized the protonation state of titratable residues. Random conformations of the ZW4864 ligand were generated through random rigid‐body rotations followed by simulated annealing. Each conformation underwent final energy minimization to refine its orientation and interactions within the β‐catenin binding cavity. Docking performance was assessed using the CDOCKER Interaction Energy, where more negative values indicate stronger binding affinity. The top‐scoring ZW4864–β‐catenin complex was further analyzed for hydrogen‐bonding, hydrophobic, and electrostatic interactions.

### 2.19 Protein–protein docking of β‐catenin with BCL9 and BCL9L

Protein–protein docking studies were performed to investigate the interaction of β‐catenin with its transcriptional coactivators BCL9 and BCL9L, and to compare their binding interfaces with that of ZW4864. The docking analyses were carried out using DS2022 with the ZDOCK, Process Poses, and RDOCK protocols. This approach was adopted to ensure the execution of comprehensive and high‐accuracy docking [63, 64, 65].

The crystal structure of β‐catenin in complex with BCL9 (PDB ID: 2GL7) was obtained from the PDB. The β‐catenin chain was extracted from this complex and used as the receptor structure and BCL9 was used as the ligand peptide. For the β‐catenin‐BCL9L interaction, the BCL9L peptide coordinates were retrieved from PDB ID: 2XB1. Protein preparation included the removal of crystallographic water molecules, correction of missing atoms, optimization of hydrogen‐bond geometries, and assignment of appropriate atom types using the Prepare Protein module in DS2022.

Rigid‐body docking was conducted using ZDOCK, which predicts protein–protein complexes by evaluating shape complementarity, electrostatic interactions, and desolvation energies through a fast Fourier transform‐based algorithm. The resulting 2000 poses were filtered and clustered using the Process Poses module to identify docking conformations localized to the known coactivator‐binding region.

Refinement and reranking of poses were performed using RDOCK, which applies the CHARMm force field to minimize steric clashes and optimize electrostatic and polar interactions. Binding affinities were calculated using the E_RDOCK scoring function, which combines electrostatic and desolvation energy components.

The best‐scoring docking poses for the β‐catenin–BCL9 and β‐catenin–BCL9L complexes were selected and compared with those of the β‐catenin–ZW4864 complex. Residue‐level interface mapping was performed to identify overlapping contact residues, providing structural evidence for ZW4864's potential competitive inhibition at the coactivator‐binding pocket of β‐catenin.

### 2.20 Off‐target analysis of ZW4864 drug using ligand profiler

The off‐target profiling of ZW4864 was performed using the *Ligand Profiler* protocol implemented in the Biovia Discovery Studio software suite 2022 (DS2022). This protocol maps a query compound onto a collection of over 250 K pharmacophore models in the PharmaDB database, which are derived from experimentally validated protein‐ligand complexes. In total, 368 pharmacophore models were screened for ZW4864. Of these, 204 pharmacophore models corresponded to 78 unique protein targets. The top 10 predicted off‐targets of ZW4864, ranked in decreasing order of Fit‐Value, are listed in Table S2. The Fit‐value reflects the geometric and chemical complementarity between ZW4864 and each pharmacophore model, with a higher value indicating stronger alignment and a higher likelihood of molecular recognition.

### 2.21 Data and statistical analysis

The analysis of the data was conducted using Microsoft Excel 2021, and the results are expressed as the mean ± standard deviations. Statistical analysis was performed using the software IBM SPSS Statistics Version 30 with combined independent biological replicates. The two‐tailed unpaired Student's t‐test was utilized to compare two groups. A 95% confidence interval (*P*‐value <0.05) was considered statistically significant and marked with ‘*’ in the graphs (*P ≤ 0.05, **P ≤ 0.01, ***P ≤ 0.001).

## 3 Results

### 3.1 Stable BCL9L knockdown represses the invasion capacity of Cal29 cells in a Boyden chamber and *ex vivo* porcine bladder model

**Fig. 1:**
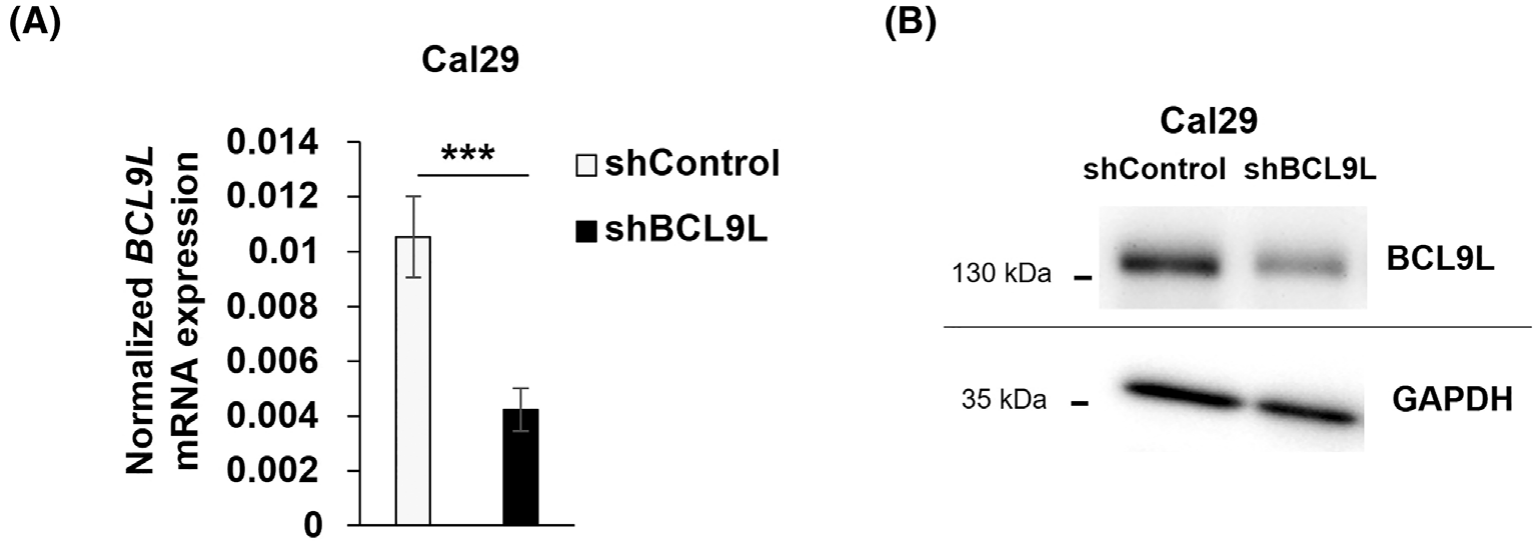
Analysis of BCL9L knockdown efficiency in Cal29 cells after lentiviral transduction. (A) mRNA expression of BCL9L after knockdown by qRT‐PCR (*n* = 3) in Cal29 cells. (B) Western blot for BCL9L protein in Cal29 cells. All western blot analyses are shown in Fig. S1. The data are expressed as the mean ± standard deviation of three experiments, and statistical analysis was performed by two‐tailed unpaired Student's *t*‐test with ***P ≤ 0.001.

Whole‐exome sequencing of patients diagnosed with progressive urothelial carcinoma revealed mutations in several genes directly or indirectly involved in Wnt/β‐catenin signaling. We have demonstrated that the knockdown of BCL9L by transient transfection of siRNA repressed the proliferation, migration and invasion of BC cell lines, thereby suggesting an oncogenic role for BCL9L in bladder cancer [38]. On the basis of these observations, we established a BC cell line with stable knockdown of BCL9L using a lentiviral transduction system to analyze the effect of BCL9L in a more complex experimental design and for a longer period of time. The mRNA expression levels of BCL9L were significantly reduced in one clone after stable knockdown compared to shControl (fold change −2.5; *P* < 0.001, Fig. 1A) and reduced protein level after knockdown was shown by western blot (Fig. 1B), confirming knockdown efficiency. The confirmed stable clone with BCL9L knockdown was used for further analysis.

**Fig. 2:**
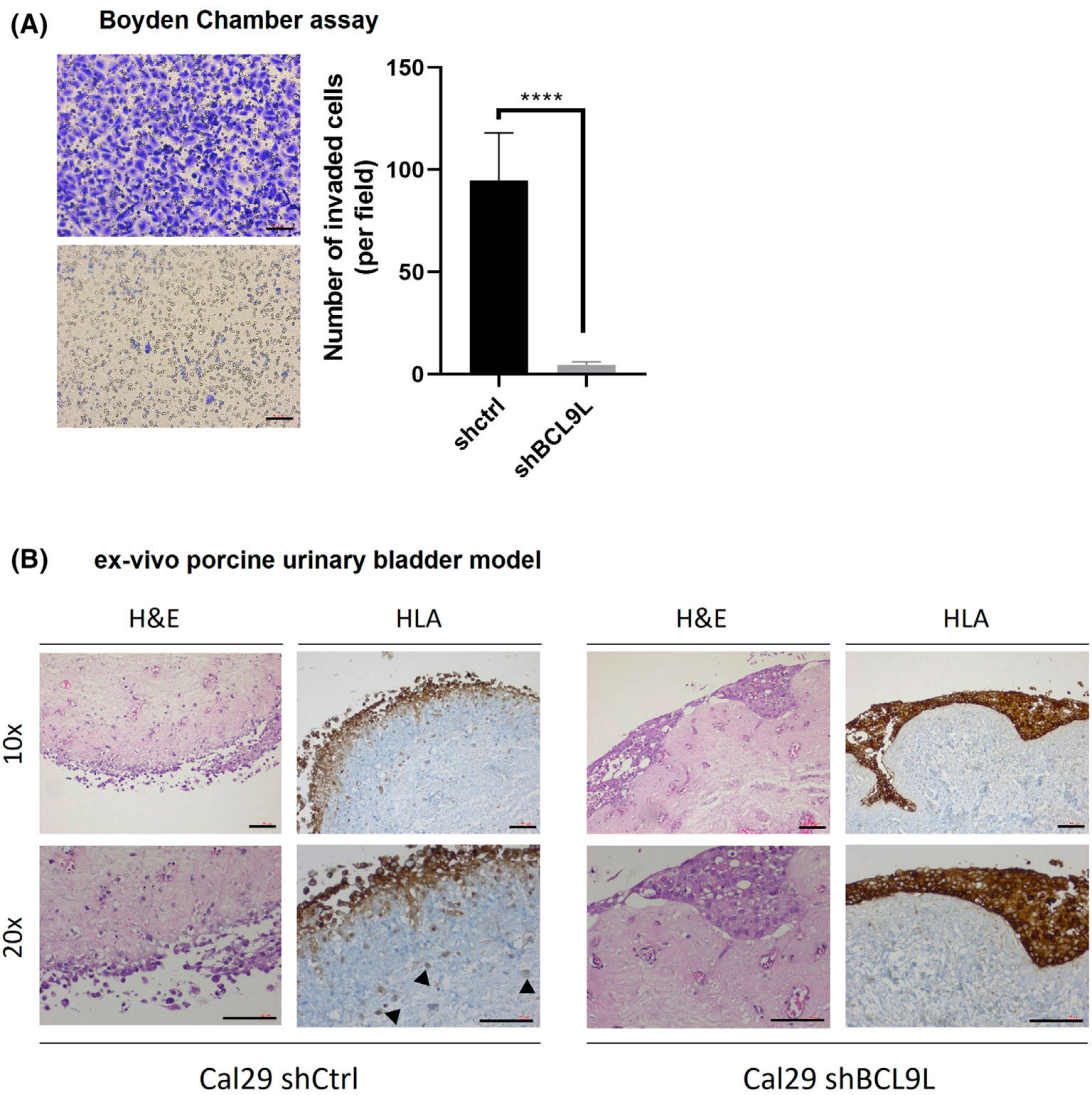
BCL9L knockdown inhibits the invasion of Cal29 cells. (A) Boyden chamber assays for stable knockdown of BCL9L in Cal29 cells stained with crystal violet. For the analysis, five different fields were included for counting the cells. The data are expressed as mean ± standard deviation and the experiment was repeated 3 times. Scale bar = 20 μm. Statistical analysis was performed by two‐tailed unpaired Student's *t*‐test with ****P ≤ 0,0001. (B) Porcine urinary bladder model. shControl and shBCL9L Cal29 cells were seeded onto de‐epithelialized bladder tissue and grown in organ culture. After 14 days, the tissue was fixed and stained with hematoxylin and eosin or an HLA antibody to detect human urothelial tumor cells. Scale bar = 100 μm.

The objective of this study was to analyze the impact of stable BCL9L knockdown on invasiveness using an *ex vivo* porcine urinary bladder model. Organ models are more suitable for understanding the invasiveness capacity of tumor cells in the whole‐organ environment. These models are designed to consider the interactions between cancer cells and the extracellular matrix as well as organ‐related stroma, thereby more effectively mimicking natural conditions [66]. First, the effect of stable BCL9L knockdown of Cal29 on invasion was analyzed by conventional Boyden chamber assay. The results demonstrated that loss of BCL9L significantly suppressed Cal29 cell migration/invasion capacity compared with that of shControl cells after 48 h (Fig. 2A), confirming our previous findings from transient siRNA transfection. Subsequently, Cal29 cells were seeded on de‐epithelialized porcine bladders, and the invasion capacity upon BCL9L knockdown was analyzed after 14 days. The Cal29‐shcontrol cells demonstrated the capacity to invade through the stromal layer, while the shBCL9L cells exhibited a complete inhibition of invasion (Fig. 2B). In addition, Cal29 shBCL9L cells formed an epithelial layer on top of the de‐epithelialized porcine bladders. In conclusion, BCL9L knockdown has been demonstrated to impede the invasive potential of Cal29 cells in both *in vitro* and *ex vivo* models.

### 3.2 Transient BCL9 knockdown reduces cell proliferation of bladder cancer cells

**Fig. 3:**
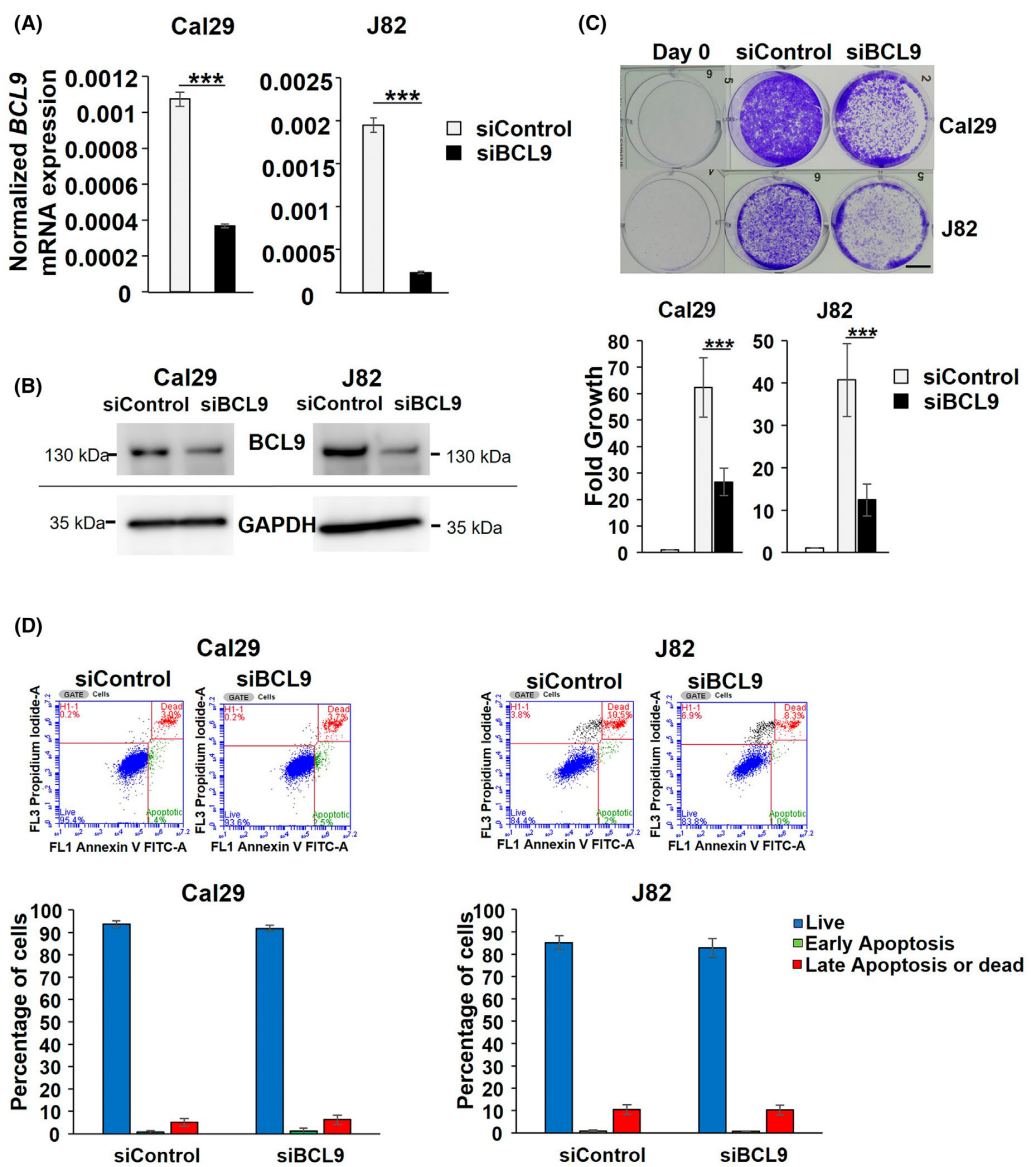
Analysis of cell proliferation and apoptosis in Cal29 and J82 cells following BCL9 knockdown. (A) mRNA expression of BCL9 after knockdown analyzed by qRT‐PCR (*n* = 3) in Cal29 and J82 cells. (B) Western blot analysis of the BCL9 protein in Cal29 and J82 cells after transfection with siBCL9. Whole western blot are shown in Fig. S2. (C) Crystal violet staining for analysis of cell proliferation after BCL9 knockdown in Cal29 and J82 cells (*n* = 5). Scale bar = 10 mm. (D) Apoptosis assay through flow cytometry was performed by dual staining with annexin V‐FITC and propidium iodide kit (*n* = 3). All date were expressed as mean ± standard deviation. ***P ≤ 0.001 two‐tailed unpaired Student's *t*‐test, *n* = independent experiments.

The present study aims to analyze the role of BCL9 (BCL9L homolog) in BC, which has not yet been examined. Here, we investigated the functional role of BCL9 in the BC cell lines Cal29 and J82 through transient knockdown experiments utilizing siRNA. The mRNA levels were significantly reduced after BCL9 knockdown in comparison with siControl (fold change −2.9 for Cal29 and − 8.6 for J82 cells, *P* < 0.001, Fig. 3A). Furthermore, the reduction in protein level was confirmed through western blot analysis (Fig. 3B), thereby validating the efficacy of the knockdown via siRNA transfection. The whole western blot image is shown in Fig. S2.

Crystal violet staining was used as an indirect method to assess cell proliferation. The knockdown of BCL9 significantly reduced cell proliferation of Cal29 and J82 cells in comparison with siControl (Fig. 3C). This finding indicates that BCL9 could play a role in promoting cell proliferation of BC cells. Subsequently, we investigated the hypothesis of whether the inhibition of cell proliferation is mediated through the induction of apoptosis. This issue was addressed by conducting a flow cytometry analysis using annexin V‐FITC and PI staining kit. Initially, Cal29 and J82 cells were treated with camptothecin (10 μm), serving as a positive control for the induction of apoptosis. Treatment of Cal29 and J82 cells with camptothecin resulted in a significant induction of apoptosis after 24 h (Fig. S3), thereby demonstrating the feasibility of utilizing this method for the detection of apoptotic cells. Notably, the knockdown of BCL9 did not induce apoptosis in Cal29 and J82 cells, as compared to the siRNA control (Fig. 3D). In conclusion, the depletion of BCL9 using siRNA resulted in decreased cell proliferation in Cal29 and J82 cells without the induction of apoptosis.

### 3.3 Transient BCL9 knockdown represses the migration and invasion of bladder cancer cells

**Fig. 4:**
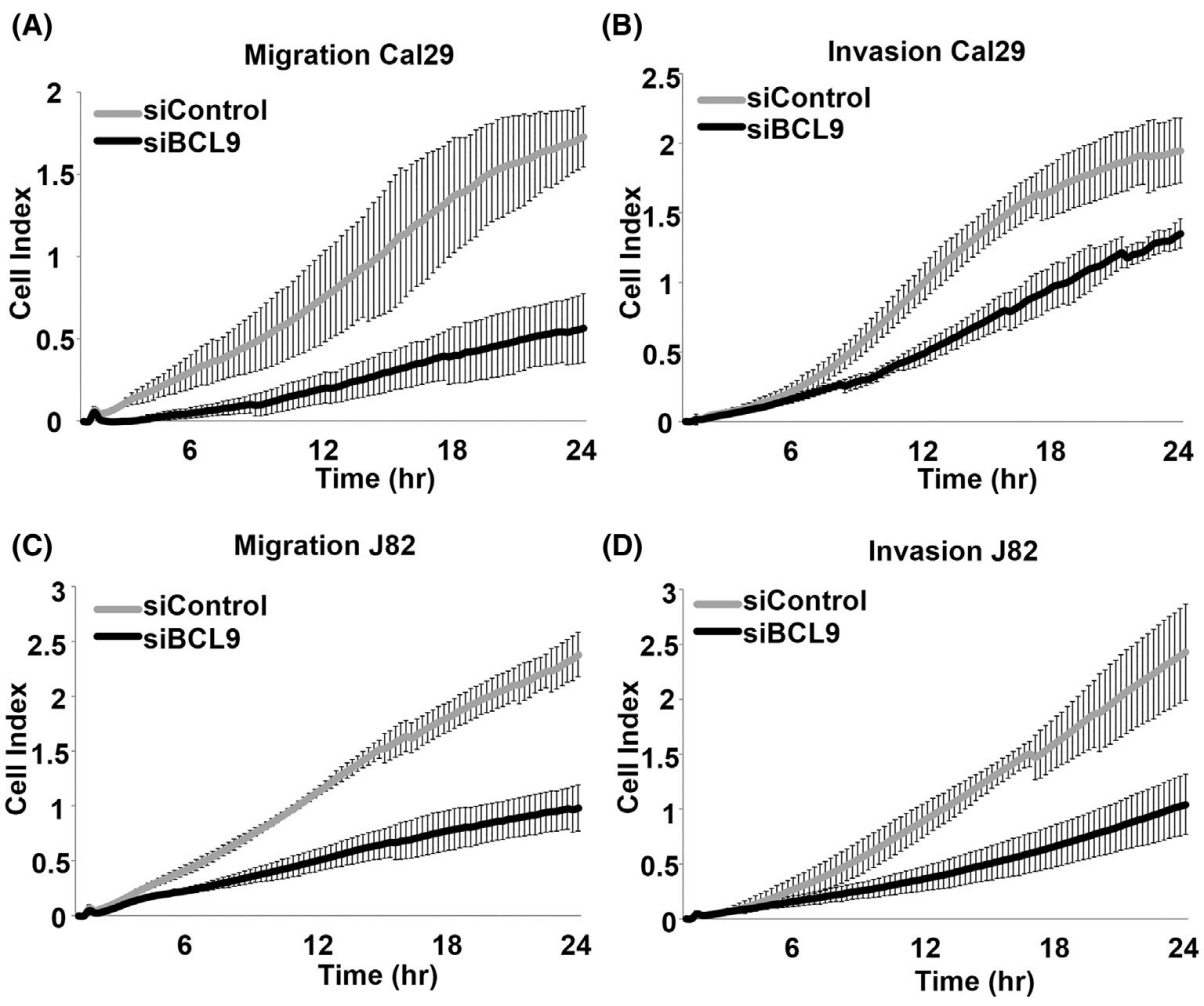
Real‐time migration and invasion analysis by xCELLigence system after BCL9 knockdown. (A) Cal29 cell migration and (B) invasion (*n* = 3). (C) Migration and (D) invasion of J82 cells (*n* = 3). For migration, 2 × 10^4^ cells pretransfected with siBCL9 or siControl were seeded into CIM plates, and migration was analyzed for 24 h. For invasion, 4 × 10^4^ pretransfected cells were seeded into CIM plates precoated with Matrigel, and invasion was analyzed for 24 h. The cell index values corresponded to the number of migrated or invaded cells. The figure represents one of the biological replicates. All three independent biological replicates are shown in Fig. S4. The data are expressed as the mean ± standard deviations of three technical replicates.

To investigate whether BCL9 exerts an influence on the migration and invasion of the BC cell lines Cal29 and J82, we conducted real‐time migration and invasion experiments using the xCELLigence RTCA system. These experiments were conducted following BCL9 knockdown by using siRNA transfection. A comparison of the BCL9 knockdown with siControl revealed the suppression of migration in Cal29 and J82 cells (Fig. 4A, C, as a representative experiment). These findings were consistent across all three independent biological replicates, as shown in Fig. S4. Furthermore, BCL9 knockdown suppressed the invasion of Cal29 and J82 cells in comparison with siControl (Fig. 4B, D, all three independent biological replicates in Fig. S4). Overall, these data suggest that BCL9 plays a critical role in promoting the migration and invasion of BC cells. In conclusion, the results showed that BCL9 could promote the proliferation, migration, and invasion of Cal29 and J82 cells, thereby emphasizing its oncogenic role in BC cells.

### 3.4 Computational and pharmacokinetic analysis predicts disruption of β‐catenin/BCL9 protein–protein interaction by the inhibitor ZW4864

Wnt/β‐catenin inhibitors have been shown to demonstrate antitumor activity in many cancer entities, thereby supporting their potential as a novel therapeutic strategy against cancer. In recent years, significant attention has been directed towards inhibitors that disrupt the interaction of β‐catenin with the coactivator protein BCL9(L). In this study, we focused on the recently discovered and commercially available small molecule ZW4864, which has been reported to disrupt the protein–protein interaction of β‐catenin with the BCL9 protein [67].

To gain deeper insights into the binding mechanism and confirm the interaction between the β‐catenin and ZW4864, molecular docking analyses were performed. In addition, the role of key residues involved in disrupting the interaction between β‐catenin and its coactivators BCL9/BCL9L was investigated. The docking results revealed a strong interaction between β‐catenin and ZW4864, with a CDOCKER interaction energy of −48.64 kcal/mol, indicating high binding affinity. The analysis identified nine bonds formed within the β‐catenin active site involving eight different amino acid residues, five of which (Leu159, Lys170, Ala171, Met174, and His176) serve as key residues contributing to ligand stabilization (Table S1). Notably, three of these residues (Leu159, Lys170, and Met174) are also critical for β‐catenin interactions with BCL9 or BCL9L, with Met174 being common to all three complexes. This overlap demonstrates that ZW4864 binds to the same coactivator‐binding interface of β‐catenin.

**Fig. 5:**
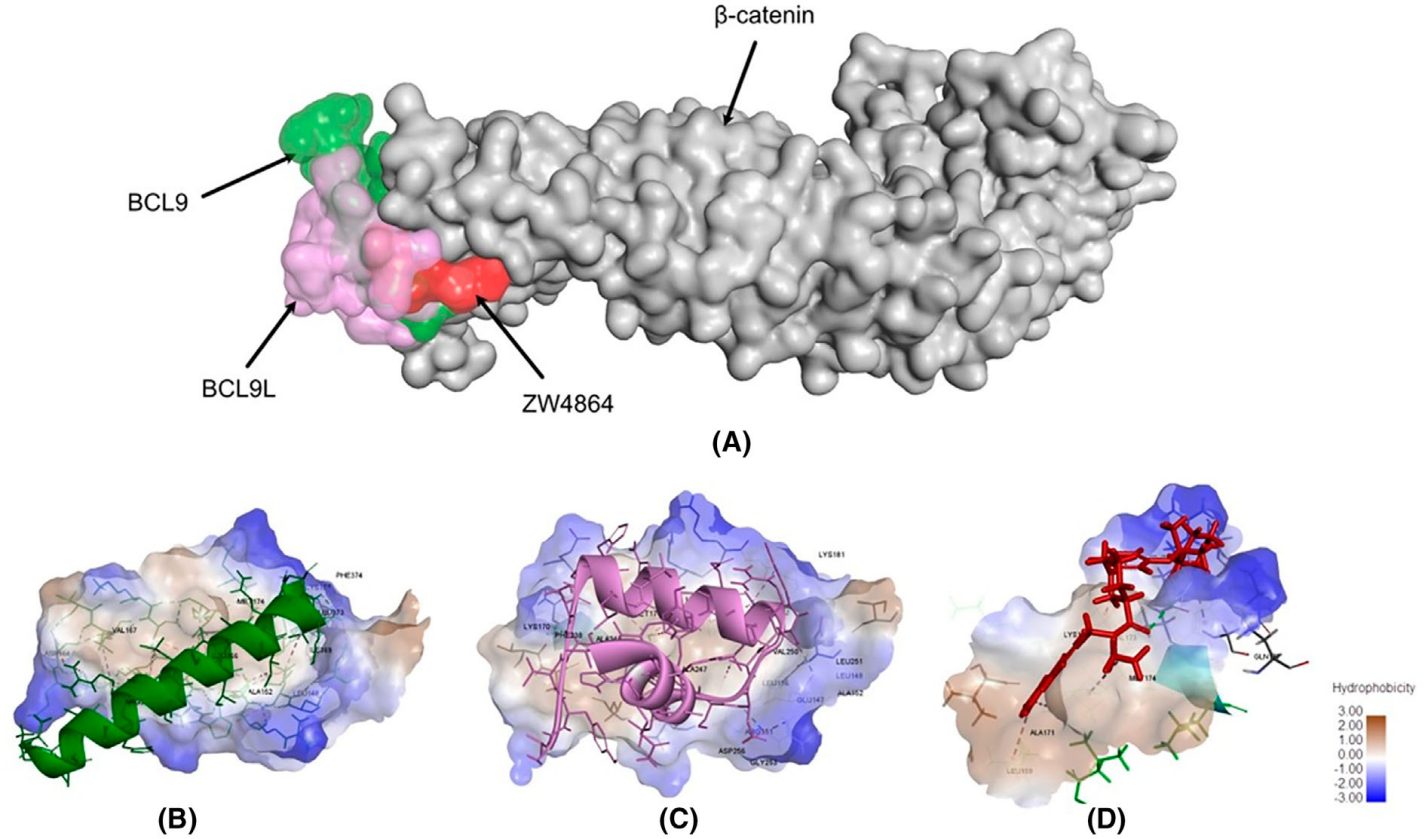
Docked complexes of β‐catenin. (A) β‐catenin is shown in gray surface model, with BCL9 (green), BCL9L (pink), and ZW4864 (red) surface models. All these ligands share the same site. depicted in green. Individual complexes are shown in (B) β‐catenin‐BCL9, (C) β‐catenin‐BCL9L, and (D) β‐catenin‐ZW4864. In individual complexes, ligands are shown as stick model, and the binding cavity of β‐catenin is highlighted as surface colored based on the hydrophobicity scale. The key amino acid residues that interact with β‐catenin are also labeled. Protein preparation was performed using the Prepare Protein module in Biovia Discovery Studio 2022.

Furthermore, the inhibitory mechanism of ZW4864 is evaluated and the interactions of β‐catenin with its coactivators BCL9 and BCL9L are characterized using the ZDOCK and RDOCK algorithms in DS2022. For BCL9L, 84 refined poses were analyzed, with Pose 12 from Cluster 1 emerging as the most favorable (E_RDOCK = −10.50 kcal/mol). For BCL9, a total of 100 refined poses were examined, with Pose 5 from Cluster 5 identified as the most stable (E_RDOCK = −22.40 kcal/mol). A comparison of the β‐catenin–BCL9/BCL9L complexes with the β‐catenin–ZW4864 complex was conducted, revealing that ZW4864 binds to the same interface used by BCL9 and BCL9L. Notably, four key residues—Leu159, Lys170, Ala171, and Met174—are frequently implicated in both protein–protein and protein–ligand interactions. Furthermore, ZW4864 exhibited a substantially higher interaction energy (−48.64 kcal/mol) compared with BCL9 (−22.40 kcal/mol) and BCL9L (−10.50 kcal/mol), suggesting a stronger and potentially competitive binding mode (Table S1; Fig. 5).

In conclusion, the findings indicate that ZW4864 could disrupt protein interaction of β‐catenin with BCL9 and BCL9L, which is facilitated by shared key residues.

### 3.5 Off‐target analysis of ZW4864 drug using ligand profiler

The off‐target profiling of ZW4864 was performed using the *Ligand Profiler* protocol implemented in the Biovia Discovery Studio software suite 2022 (DS2022). Following the identification of 10 potential off‐targets of ZW4864, including Carbonic anhydrase 1 and 2, Ferrochelatase, Cyclin‐dependent kinase 2, Bromodomain‐containing protein 4 (BRD4), Tyrosine‐protein kinase Lck, and Heat shock protein HSP90‐α, pathway enrichment analysis was conducted to elucidate their biological relevance (Tables S2 and S3). The *ShinyGo* tool was utilized to conduct gene ontology (GO) and pathway enrichment analyses, with the results being ranked according to fold enrichment values. The enriched pathways included nitrogen metabolism, prostate cancer, progesterone‐mediated oocyte maturation, Th17 cell differentiation, human T‐cell leukemia virus type 1 (HTLV‐1) infection, and metabolic pathways. Several of these pathways are critically involved in the development and progression of cancer. In particular, the enrichment of prostate cancer and Th17 cell differentiation pathways suggests the potential roles of ZW4864 targets in tumor promoting mechanisms, including inflammation, immune suppression, and angiogenesis. Furthermore, the observed enrichment of the HTLV‐1 infection pathway underscores a link to oncogenic signaling associated with T‐cell transformation.

In summary, the results of the pathway enrichment analysis indicate that ZW4864, in addition to its strong interaction with β‐catenin, may also interact with several other cancer‐associated proteins and pathways. These findings highlight its potential role in modulating oncogenic processes. This knowledge has to be considered in subsequent studies.

### 3.6 ZW4864 inhibits Wnt/β‐catenin signaling in bladder cancer cell lines

**Fig. 6:**
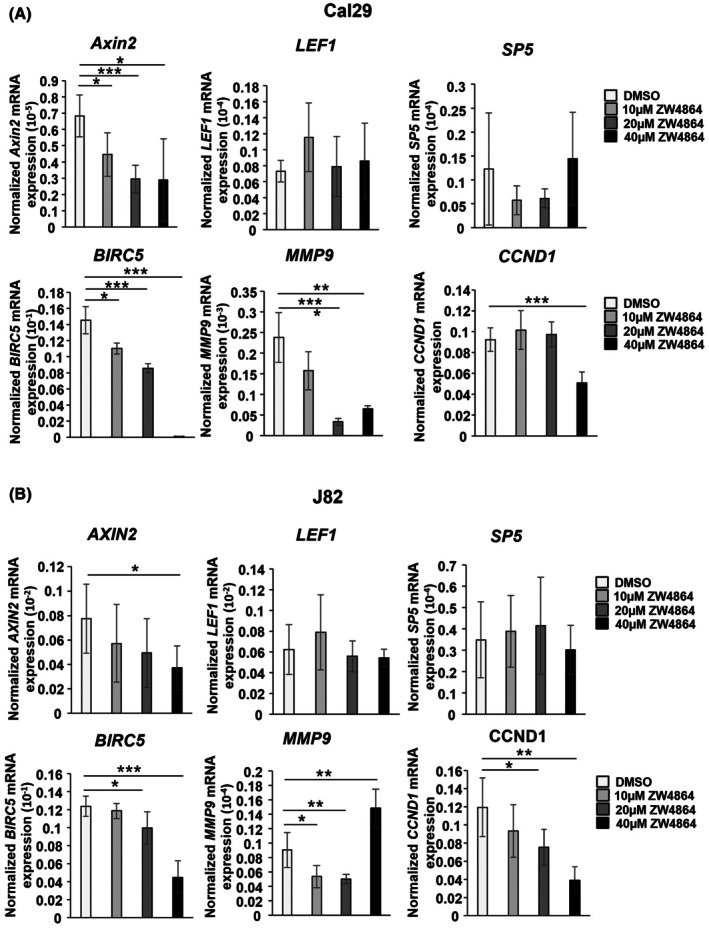
Analysis of Wnt/β‐catenin target gene expression by qRT–PCR after treatment with the BCL9/β‐catenin inhibitor ZW4864. (A) Cal29 and (B) J82 cells were treated with 10 μm, 20 μm or 40 μm ZW4864, and after 2 days, the mRNA expression of the Wnt/β‐catenin target genes AXIN2, LEF1, SP5, BIRC5, MMP9, and CCND1 was analyzed by qRT‐PCR. The mRNA expression was normalized to the housekeeping genes RPS13 and RPS23. The data are expressed as the mean ± standard deviation with *n* = 5, and statistical analysis was performed by two‐tailed unpaired Student's t‐test with *P ≤ 0.05, **P ≤ 0.01, ***P ≤ 0.001. *n*: independent biological replicates.

In accordance with the observations derived from docking experiments, which demonstrated the capacity of ZW4864 to disrupt the protein interaction of β‐catenin with the BCL9 protein, we investigated whether ZW4864 could inhibit Wnt/β‐catenin signaling in BC cell lines. Consequently, the expression of Wnt/β‐catenin target genes AXIN2, LEF1, SP5, BIRC5, MMP9, and CCND1 in response to 2 days of treatment with 10 μM, 20 μM, and 40 μM ZW4864 was analyzed in Cal29 and J82 cells by qRT‐PCR. In Cal29, the mRNA levels of AXIN2, BIRC5, and MMP9 were significantly reduced in a concentration‐dependent manner compared to the DMSO control. A significant reduction in CCND1 mRNA levels was observed in Cal29 cells treated with 40 μM ZW4864, as compared to DMSO control (Fig. 6A). In J82 cells, AXIN2, BIRC5, and CCND1 mRNA expression was significantly reduced with a more pronounced effect observed at 40 μM ZW4864 compared to the DMSO control. The level of MMP9 mRNA was found to be significantly reduced in response to treatments with ZW4864 at concentrations of 10 μm and 20 μm. However, the MMP9 mRNA level in the 40 μm ZW4864 treatment exhibited an unexpected increase compared with that in the DMSO control, for reasons that are not yet clear (Fig. 6B). Overall, the inhibitor ZW4864 demonstrated the capacity to suppress a specific subset of Wnt/β‐catenin target genes in Cal29 and J82 cells, suggesting that it inhibits Wnt/β‐catenin signaling in BC cells.

### 3.7 The inhibitor ZW4864 suppresses cell proliferation in BC cells and induces apoptosis at relatively high concentrations

**Fig. 7:**
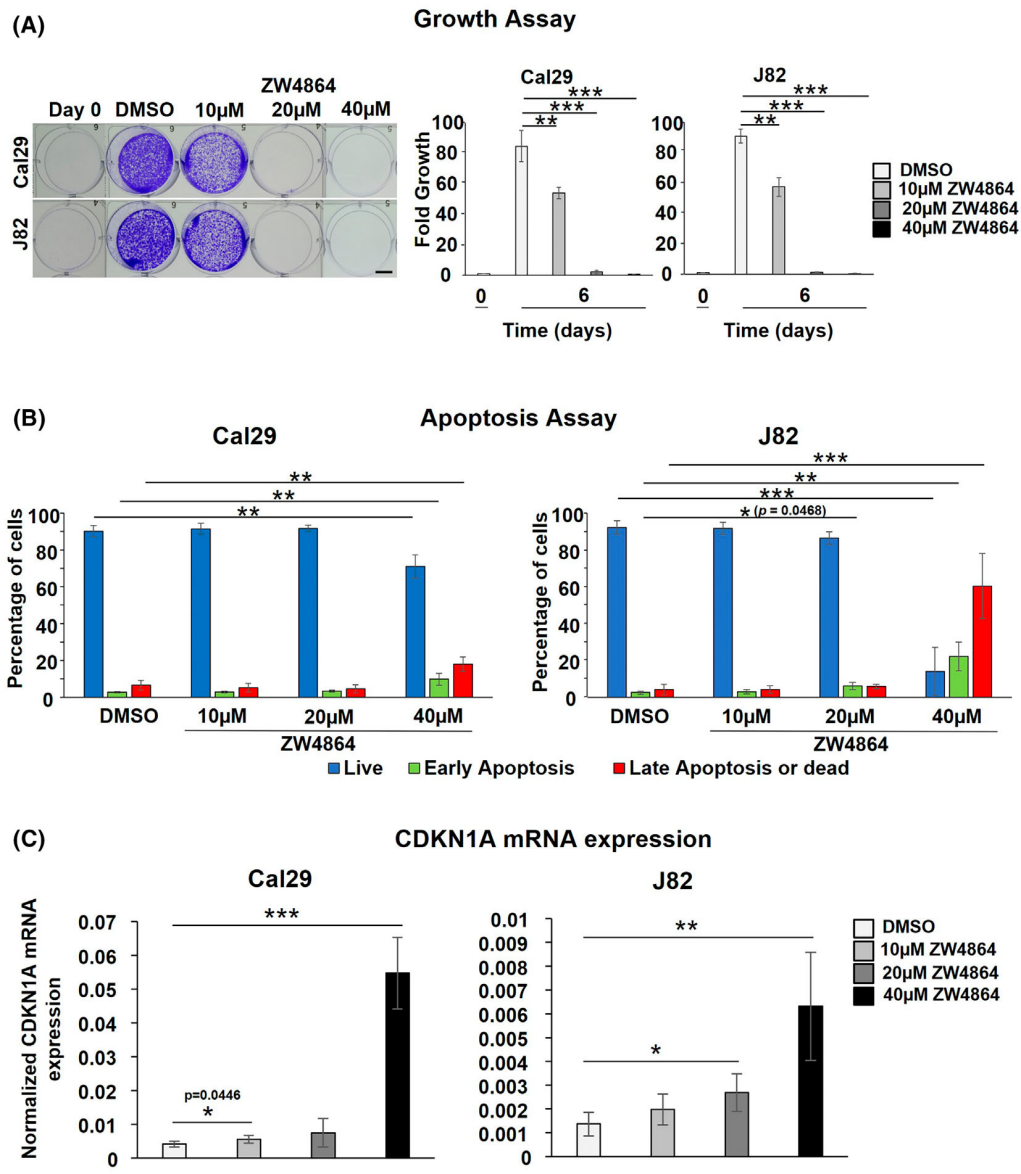
Cell proliferation and apoptosis analysis after treatment with the BCL9/β‐catenin inhibitor ZW4864. (A) Cell proliferation was analyzed by crystal violet staining of Cal29 and J82 (*n* = 5) cells after 6 days of treatment with 10 μm, 20 μm or 40 μm ZW4864. Day 0 was arbitrarily set to 1. Scale bar = 10 mm. (B) Apoptosis was analyzed by flow cytometry using dual staining with an annexin V‐FITC and propidium iodide (PI) kit. Cal29 and J82 cells (*n* = 4) were treated with 10 μm, 20 μm or 40 μm ZW4864 and analyzed after 2 days. Flow cytometry blots are available in supplementary Fig. S3B. (C) mRNA expression analysis of CDKN1A (p21 ^CDKN1A^) after 10 μm, 20 μm and 40 μm ZW4864 treatment in Cal29 and J82 cells (*n* = 5). The mRNA expression was normalized to the housekeeping genes RPS13 and RPS23. The data are expressed as the mean ± standard deviation with *n* = 5 and statistical analysis was performed by two‐tailed unpaired Student's t‐test with *P ≤ 0.05, **P ≤ 0.01, ***P ≤ 0.001. n: independent biological replicates.

Subsequent to confirming the inhibitory activity of ZW4864 on Wnt/β‐catenin signaling, we aimed to investigate whether ZW4864 has any functional effect on BC cell lines. The assessment of cell proliferation was conducted in a monolayer model using crystal violet staining of Cal29 and J82 cells following treatment with ZW4864 at a concentration of 10 μm, 20 μm, or 40 μm. In comparison with the DMSO control, ZW4864 treatment led to a significant reduction in the proliferation of both Cal29 and J82 cells after 6 days of treatment in a dose‐dependent manner (Fig. 7A). Subsequently, the impact of ZW4864 on apoptosis was investigated through the use of flow cytometry following 2 days of ZW4864 treatment. ZW4864 at concentrations of 10 μm and 20 μm did not induce apoptosis in either the Cal29 or J82 cell line (Fig. 7B, suppl. Fig. S3B). However, ZW4864 significantly induced early apoptosis and cell death at 40 μM in both cell lines (Fig. 7B, suppl. Fig. S3B). To further confirm the effect on apoptosis, the analysis of the CDKN1A (p21 CDKN1A) mRNA level was conducted. The p21CDKN1A protein has been demonstrated to play a role in the regulation of cellular growth and programmed cell death (apoptosis) mechanisms. It has been shown that overexpression of the p21CDKN1A protein could induce apoptosis in cancer cells [68]. In comparison with the DMSO control, ZW4864 (40 μm) demonstrated a significant increase in CDKN1A mRNA levels in Cal29 and J82 cells (Fig. 7C). In conclusion, the inhibition of Wnt/β‐catenin signaling by ZW4864 inhibits cell proliferation in a concentration‐dependent manner, while at higher concentrations, ZW4864 induces apoptosis in BC cells.

### 3.8 ZW4864 inhibits the migration and invasion of BC cells

**Fig. 8:**
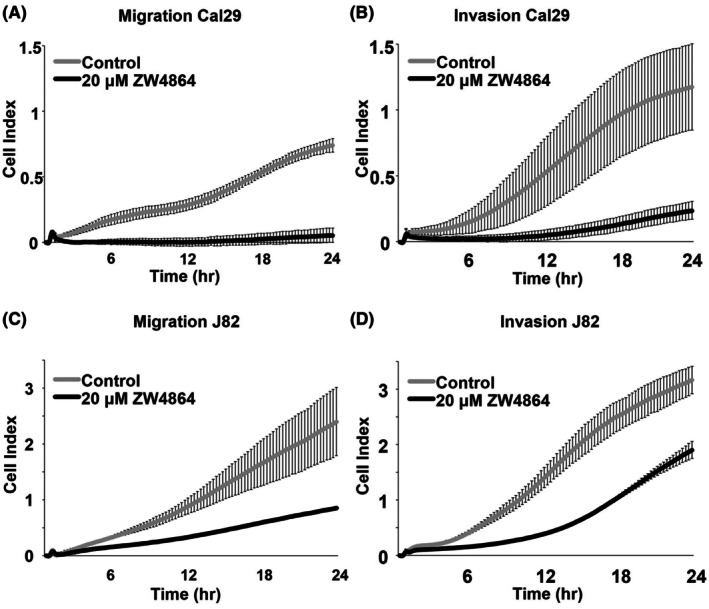
Migration and invasion analyses after treatment with the BCL9/β‐catenin inhibitor ZW4864 using the xCELLigence RTCA system. (A) Migration and (B) invasion of Cal29 cells (*n* = 3). (C) Migration and (D) invasion of J82 cells (*n* = 3). For the migration assay, 2 × 10^4^ Cal29 and J82 cells pre‐treated for 2 days with 20 μm ZW4864 or 0.1% (v/v) DMSO as a control were analyzed for 24 h. For the invasion assay, 4 × 10^4^ Cal29 and J82 cells were pretreated with 20 μm inhibitor, seeded into CIM plates precoated with Matrigel and analyzed. The cell index values correspond to the number of migrated or invaded cells. The figure represents one of the biological replicates. All three independent biological replicates are shown in Fig. S5. The data are expressed as the means ± standard deviations of three technical replicates.

Given the antiproliferative response of ZW4864 in Cal29 and J82 cells observed in previous monolayer experiments, the present study investigated the effect of ZW4864 on migration and invasion using the xCELLigence RTCA system. For the migration and invasion experiments, 20 μm ZW4864 was used because this concentration had an effect on proliferation without induction of apoptosis in the 2D monolayer experimental setup. In comparison with the DMSO control, the 20 μm ZW4864 treatment was found to inhibit the migration of both the Ca29 and J82 cell lines (Fig. 8A, C). Furthermore, ZW4864 demonstrated an inhibitory effect on the invasion of Cal29 and J82 cells in comparison with DMSO control (Fig. 8B, D). All three independent biological replicate experiments are shown in Fig. S5. The findings indicate that that ZW4864 negatively affects the migration and invasion of BC cells. In conclusion, the results show that the inhibition of the Wnt/β‐catenin/BCL9 signaling axis by the specific inhibitor ZW4864 has the potential to induce an antitumor response in BC cells.

### 3.9 ZW4864 negatively affects the growth of bladder cancer spheroids

**Fig. 9:**
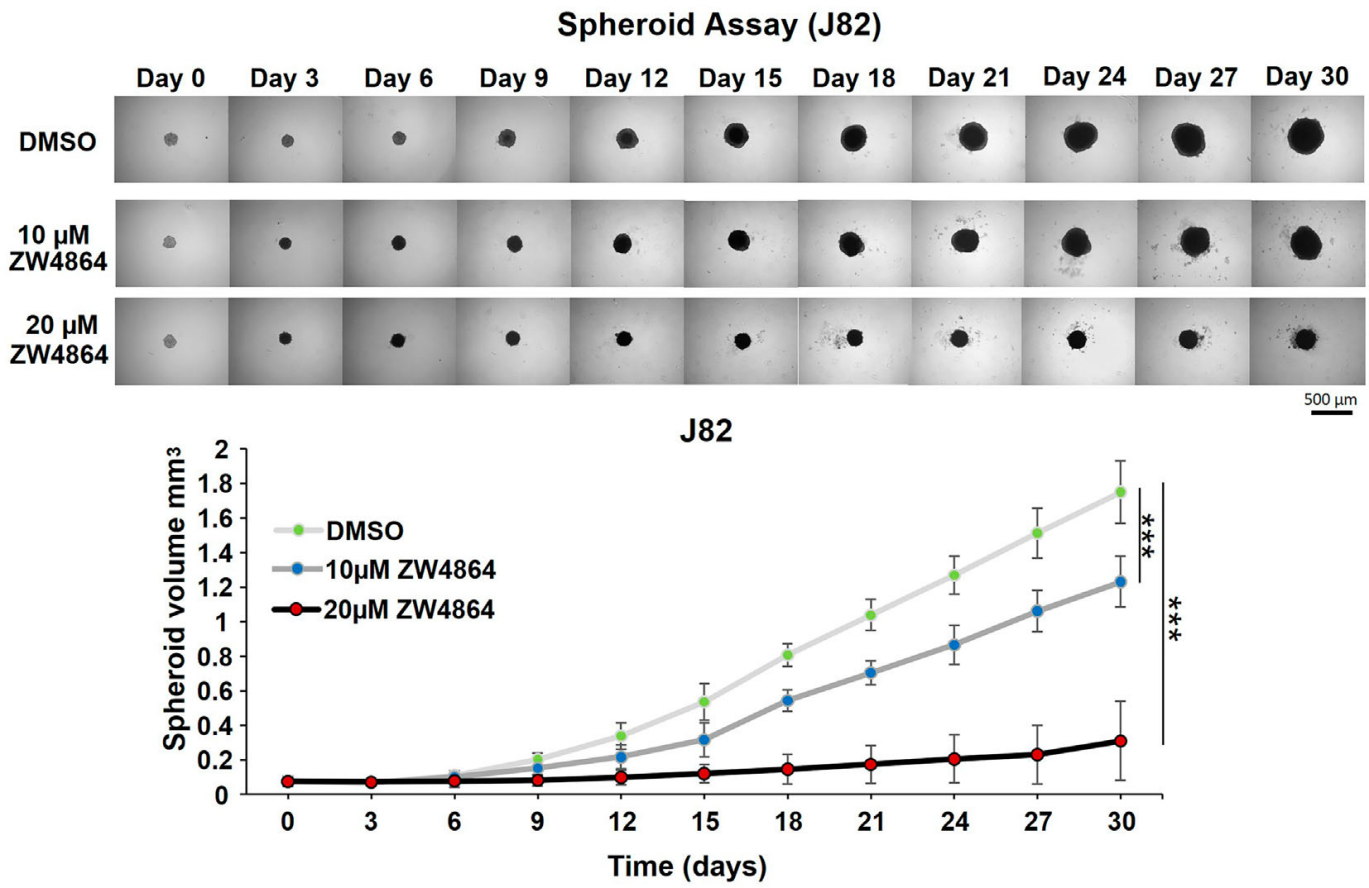
Spheroid growth analysis of J82 cells after treatment with the BCL9/β‐catenin inhibitor ZW4864. One thousand J82 cells were seeded in U‐bottom cell repellent surface 96‐well microplate to form spheroids. The spheroids were treated with 10 μm or 20 μm of the inhibitor ZW4864, compared to 0.1% (v/v) DMSO control. Scale bar = 500 μm. Day 0 was arbitrarily set to 1. The data are expressed as the mean ± standard deviation with *n* = 24, and statistical analysis was performed by two‐tailed unpaired Student's *t*‐test with ***P ≤ 0.001. *n*: number of independent spheroids.

This study demonstrated that the β‐catenin/BCL9(L) inhibitor ZW4864 suppressed the proliferation of BC cells in 2D monolayer models. It is noteworthy that this effect is mediated through the induction of apoptosis at elevated concentrations. Subsequently, the influence of ZW4864 on 3D spheroid growth and in cell‐based multicellular BC organoid models was analyzed as these models more accurately mimic the cell composition present within the tumor environment. In the spheroid experiments, J82 cells were seeded in low‐attachment cell culture plates to form spontaneous 3D spheroid structures, and the following day, the cells were treated with 10 μm, 20 μm, or 40 μm ZW4864, including DMSO used as a control. Overall, J82 spheroids demonstrated a gradual and consistent growth pattern over 30 days, making them more appropriate for long‐term drug assay analysis by inhibitors. Treatment with ZW4864 at concentrations of 10 μm and 20 μm resulted in a dose‐dependent decrease in the volume of J82 spheroids. Notably, 20 μm ZW4864 nearly completely inhibited spheroid growth compared to the DMSO control (Fig. 9). Interestingly, the spheroids treated with 40 μm ZW4864 exhibited signs of dissociation and died after 3 days; therefore, further analysis was not possible (data not shown). These data fully confirmed the results previously obtained from the 2D monolayer experiment. The effect of ZW4864 on Cal29 spheroids could not be analyzed because this cell line does not form stable spheroids over time under the described protocol.

### 3.10 ZW4864 negatively affects BC cell growth in self‐organizing organoids and completely disrupts tumor multilayer in pre‐formed BC organoids

**Fig. 10:**
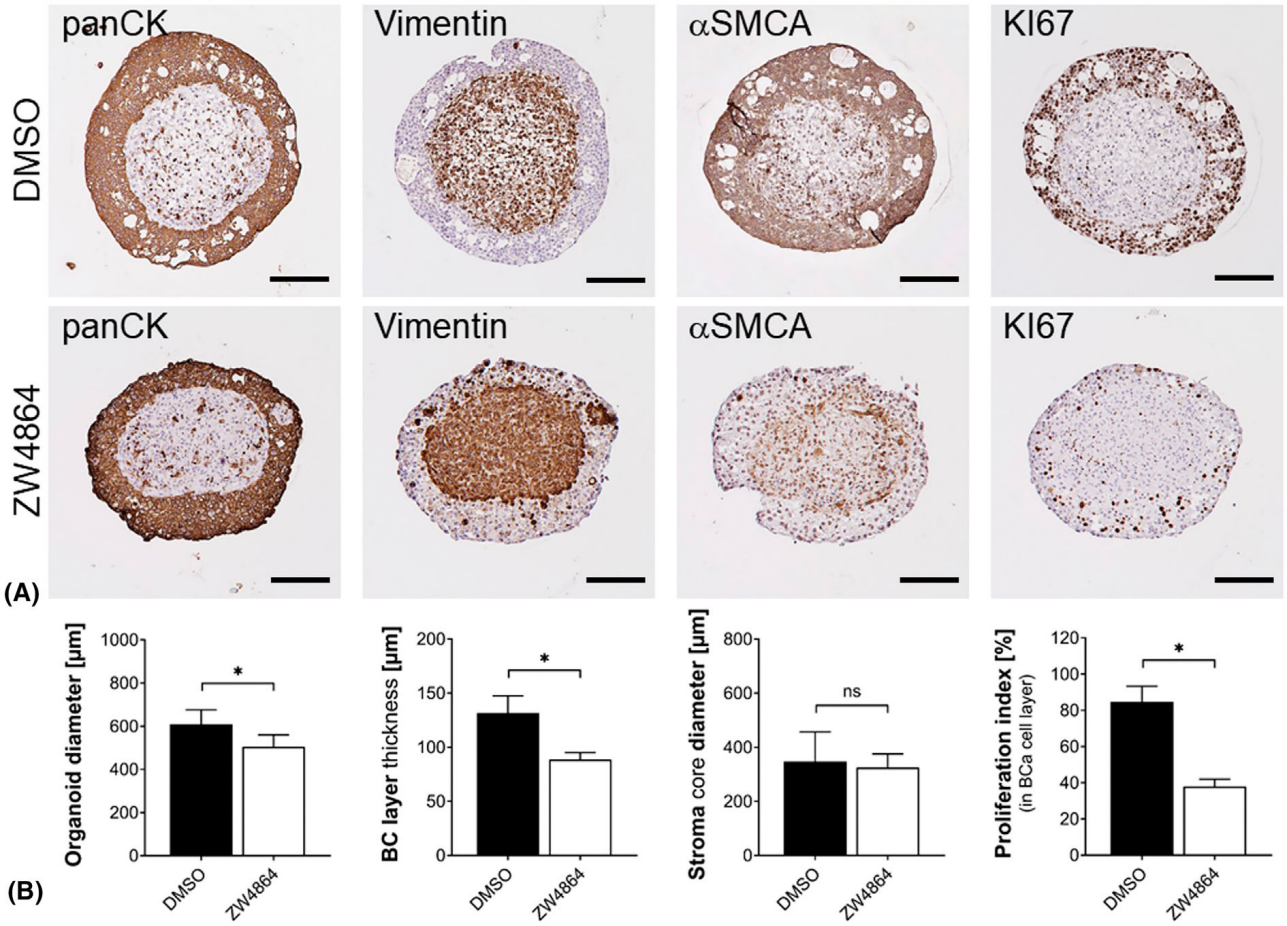
Effects of the BCL9/β‐catenin inhibitor ZW4864 on the tumor growth and self‐organization of multicellular Cal29/hBF/hBSMC organoids. ZW4864 (20 μm) was added directly for organoid generation (day 0); the organoids were fixed after 4 days of organoid culture. (A) Representative images of Cal29 organoids (ZW4864 on day 0, vs. DMSO) immunostained for panCK, vimentin, αSMCA, and Ki67. Scale bar = 150 μm. (B) Quantification of organoid size, BC layer thickness, the diameter of the stroma cell core, and the proliferation index. BCL9L inhibition on day 0 resulted in significantly decreased organoid diameter, thickness of the tumor cell layer, and tumor cell proliferation, while the stroma cell core was not affected; * significant differences compared with the vehicle control (DMSO); *P* ≤ 0.05, Mann–Whitney test, mean + SD.

This study was conducted to investigate the effects of ZW4864 on the formation and self‐organization of organoids consisting of Cal29 bladder cancer tumor cells, human bladder fibroblasts (hBFs), and human bladder smooth muscle cells (hBSMCs). For this purpose, the inhibitor was added directly into the mixed cell suspension on the day of organoid generation (day 0), and the suspension was cultured for a period of 4 days. Furthermore, the formation of inverted bladder‐like organoid structures was observed, with an outer tumor layer that was positive for pan‐cytokeratin, surrounding a core of fibroblasts and α‐smooth muscle cells that were positive for vimentin and α‐smooth muscle actin, even under ZW4864 treatment (Fig. 10A). However, the addition of ZW4864 resulted in a substantial reduction in BC layer thickness and BC cell proliferation (Ki67), while the diameter of the stromal cell core was not affected (Fig. 10B).

**Fig. 11:**
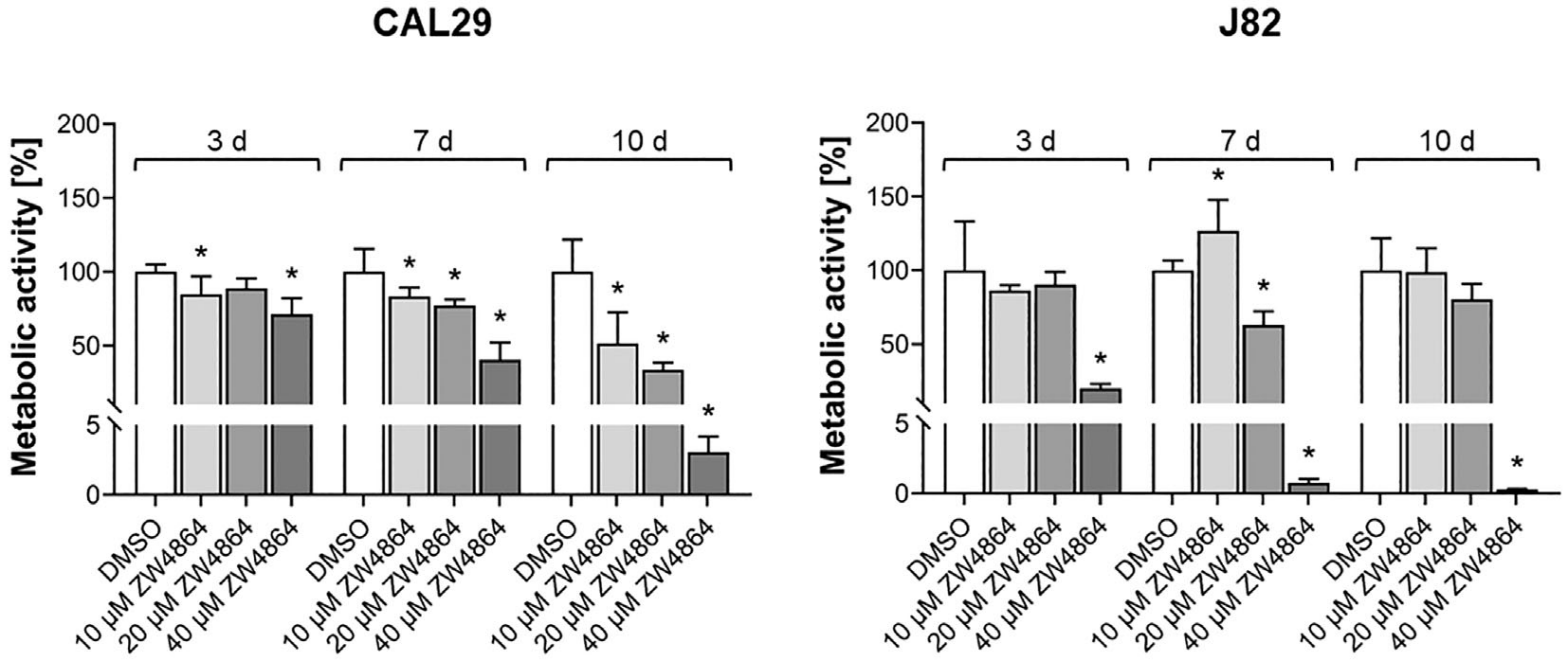
Viability assay in pre‐formed (multilayered) Cal29 and J82 organoids after treatment with the BCL9/β‐catenin inhibitor ZW4864. The inhibitor was added after 3 days of organoid culture. Bulk analysis of metabolic activity by CellTiter‐Glo^®^ 3D cell viability assay 3, 7, and 10 days after ZW4864 addition. Significantly decreased viability was observed in a concentration‐dependent manner in all spheroids; * significant differences compared to vehicle control (DMSO); *P* ≤ 0.05, One‐way ANOVA, mean + SD.

Subsequently, the focus shifted to the analysis of ZW4864's impact on cell viability. For this purpose, J82 or Cal29 cells were co‐seeded with hBF and hBSMC cells to form spontaneous 3D organoid structures. The pre‐formed multicellular BC organoids were treated after 3 days with 10 μm, 20 μm, or 40 μm ZW4864. The cell viability of the organoids was analyzed through live‐cell imaging over a 10‐day period and by end‐point measurements of metabolic activity using the CellTiter‐Glo^®^ 3D assay after 3, 7, and 10 days of treatment with ZW4864. Live‐cell imaging revealed that ZW4864 treatment resulted in disintegration and dissociation of BC organoids, especially at 40 μm ZW4864, after 7 and 10 days of treatment (Fig. S6). Furthermore, ZW4864 significantly reduced the metabolic activity of both J82‐based and Cal29‐based organoids. The data from J82 organoids align with the response observed in J82 spheroids. Treatment with 20 μm ZW4864 resulted in a significant reduction in organoid viability, whereas 40 μm ZW4864 led to nearly complete organoid death after 7 to 10 days. Conversely, concentration‐dependent effects were observed in Cal29 organoid cultures (Fig. 11).

**Fig. 12:**
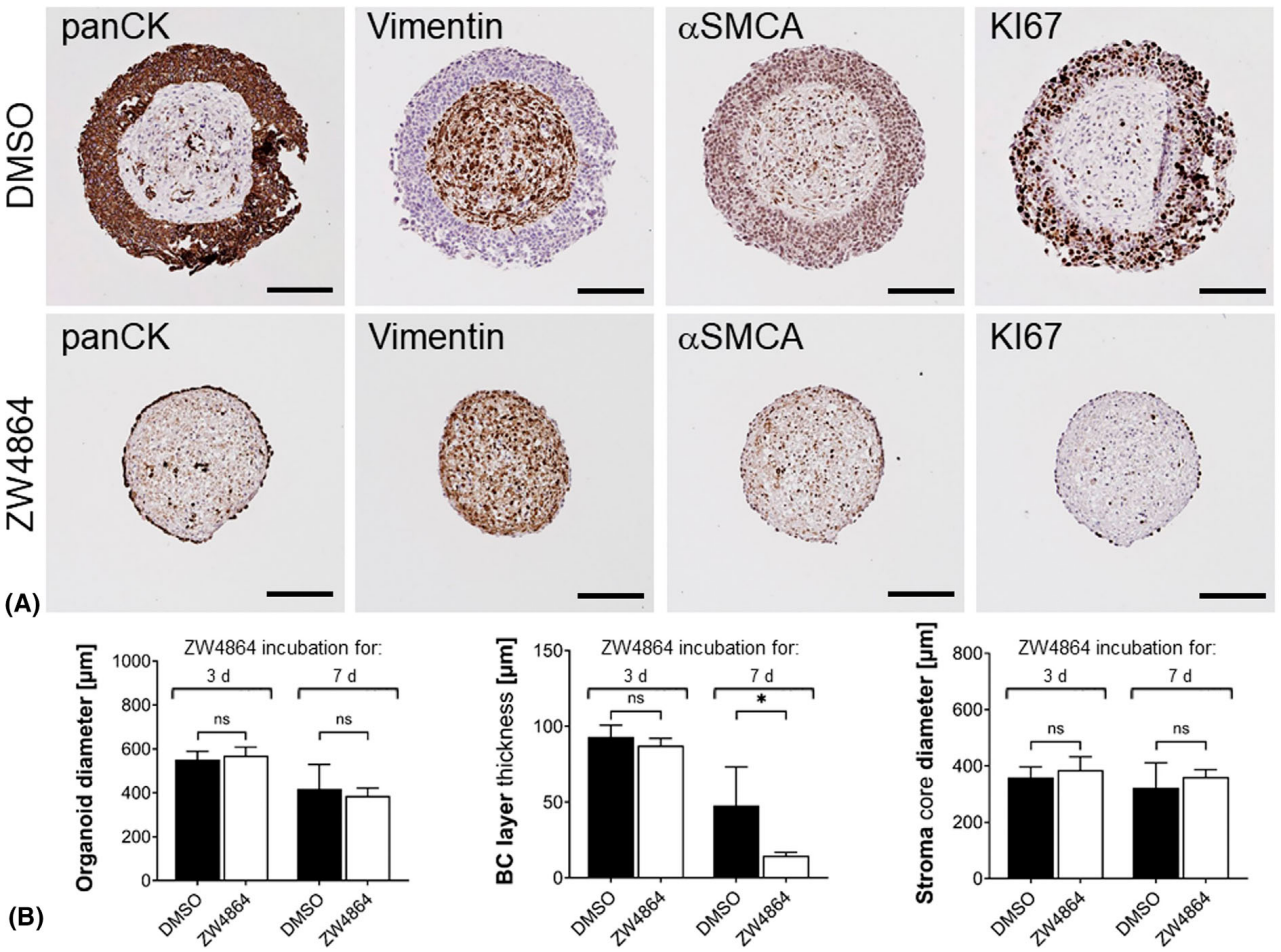
Tumor‐selective effects of the inhibitor ZW4864 in pre‐formed (multilayered) Cal29 organoids. The inhibitor ZW4864 (20 μm) was added after 3 days of organoid culture; the organoids were fixed after 3 and 7 days of culture. (A) Representative images of Cal29 organoids (ZW4864 vs. DMSO) immunostained for panCK, vimentin, αSMCA, and Ki67 after 7 days of ZW4864 treatment. Scale bar: 150 μm. (B) Quantification of organoid size, BC layer thickness, and the diameter of the stroma cell core. BCL9(L)/β‐catenin inhibition resulted in almost complete detachment of the tumor layer after 7 days, while the stromal cell core was not affected; * significant differences compared to vehicle control (DMSO); *P* ≤ 0.05, Mann–Whitney test, mean + SD.

The tumor‐selective effects of the inhibitor ZW4864 on pre‐formed BC organoids were further studied in histologically processed samples. Three‐day‐old Cal29 and J82 organoids were treated with 20 μM ZW4864 for 3, 7, and 10 days, and immunostaining was performed to analyze organoid structure (panCK, vimentin, αSMCA, Ki67). Representative images of Cal29 organoids are shown in Fig. 12. Treatment with ZW4864 resulted in the nearly complete eradication of the outer Cal29 tumor cells after 7 days, while the stroma cell core remained (Fig. 12A). The thickness of the BC tumor cell layer significantly decreased from 48 μm to 15 μm. However, no alterations were observed in the stroma core diameter (Fig. 12B). The quantification of Cal29 tumor cell proliferation was not possible due to the absence of the BC cell layer. However, analysis of Cal29 cultures after 10 days was not feasible due to significant organoid dissociation. The effects in J82 were less prominent; however, even at present, treatment with 20 μm ZW4864 led to a significant reduction in the thickness of the tumor layer after 10 days. The stroma core demonstrated no alterations (Fig. S7).

## 4 Discussion

Bladder cancer (BC) is the most prevalent malignant tumor in the urinary system and is the ninth most common cancer worldwide [1]. BC is a highly heterogeneous disease that often leads to metastasis and poor overall survival [9, 10, 69, 70, 71]. Despite BC's high progression rate, it remains an understudied tumor entity, and the molecular mechanisms responsible for BC progression are not well understood. In order to effectively combat BC, it is important to identify specific targets that are directly associated with its progression. An investigation of these critical tumor drivers will provide further strategies for BC therapy. As indicated in our previous whole‐exome sequencing study, we identified several tumor‐associated targets to be mutated in progressive BC [57]. A subsequent investigation into the mutated genes revealed an overrepresentation of the Wnt/β‐catenin signaling pathway. Specifically, single point mutations were identified within the regulatory 5′ and 3′ UTR regions of BCL9L, a coactivator of β‐catenin. We demonstrated that a mutation at the 3′ UTR could reduce gene expression using a luciferase activity reporter assay in comparison to the wild‐type. These finding suggested that mutations in the UTR could be a potential mechanism for regulating BCL9L expression in bladder cancer. Furthermore, BCL9L protein expression was found to be predominantly higher in muscle‐invasive BC (MIBC) patients compared to non‐muscle‐invasive BC (NMIBC) patients. Moreover, our previous study have demonstrated that the inhibition of Wnt/β‐catenin signaling and transient BCL9L knockdown exert a negative effects on the proliferation, migration and invasion of BC cell lines [38]. We previously suggested that Wnt/β‐catenin signaling and its coactivator BCL9L have an oncogenic effect on BC progression. In this study, we conducted a further investigation into the functional analysis of BCL9L using stable knockdown Cal29 cells in an *ex vivo* porcine organ model. BCL9 has been identified as the only known homolog of BCL9L; however, its function in BC progression remains unclear. Our previous study failed to detect mutations in BCL9. It has been demonstrated that both BCL9 and BCL9L possess analogous functions, acting as coactivators of β‐catenin. These both proteins are characterized by the shared of a conserved HD2 domain, which is responsible in mediating interaction with β‐catenin, thereby activating Wnt/β‐catenin signaling [39, 40, 41]. Therefore, an initial objective of this study was to investigate the functional role of transient knockdown of BCL9 in BC using Cal29 and J82 cells, spheroids and organoid models. A secondary objective was to investigate the impact of the small molecule inhibitor ZW4864, a promising pharmaceutical agent on bladder cancer. ZW4864 functions as a small inhibitor, thereby hindering the interaction of β‐catenin with both BCL9 and BCL9L coactivators. This interaction could play critical role in the progression of bladder cancer.

The expression of BCL9L has been found to be significantly correlated with the progression of various cancer types, including breast cancer, colorectal cancer, hepatocellular carcinoma and other oncological diseases [45, 56, 72, 73]. However, BCL9L has not been the focus of extensive research in BC. In 2022, we demonstrated for the first time that BCL9L exhibits a heterogeneous expression pattern in BC, with higher expression levels observed in MIBC compared to NMIBC and normal‐like urothelium [38]. Our previous study offered preliminary indication that BCL9L expression to be associated with BC tumor stage and progression. A limitation of the study was the small number of patients in the cohort. Moreover, transient knockdown of BCL9L by siRNA transfection inhibited the proliferation, migration, and invasion of BC cell lines in a 2D monolayer model, thereby suggesting an oncogenic role for BCL9L in BC [38]. This finding aligns with the results of other studies on various tumor entities, in which BCL9L was thoroughly examined. These studies utilized knockdown and overexpression experiments to demonstrate the oncogenic effect of BCL9L in different cancer cell [45, 53, 55, 56, 72, 73, 74]. However, it has to be emphasized that our previous work is the only study that associated BCL9L with BC progression. In accordance with these findings, our subsequent focus was on further analysis of BCL9L analysis in an *ex vivo* porcine bladder model. We demonstrated that BCL9L knockdown inhibits the invasion of BC cells in an *ex vivo* porcine model, further verifying the oncogenic activity of BCL9L in bladder cancer. Such organ models are more effective for a comprehensive understanding of the invasive potential of tumor cells in incorporating the complex environment of an entire organ. These models effectively consider the complex interactions between cancer cells with the extracellular matrix and the organ‐specific stroma. Consequently, they offer a more accurate representation of the tumor microenvironment conditions. These models are essential for enhancing our comprehension of cell–cell communication in tumor microenvironment, leading to more effective cancer research and treatment strategies [66].

BCL9 and BCL9L have been shown to perform analogous functions, and the tumor driver role of BCL9 in cancer progression has been validated for multiple cancers. BCL9 is overexpressed in various tumor types and is associated with cancer progression and poor prognosis. Furthermore, BCL9 has been found to overexpressed in hepatocellular carcinoma, underscoring its potential as a vital prognostic marker for this aggressive cancer [47]. A significant proportion of patients diagnosed with colorectal cancer show high level of BCL9, suggesting that BCL9 plays a crucial role in the development and progression of colorectal cancer, predominantly through its involvement in the Wnt/β‐catenin signaling pathway. Elevated levels of BCL9 appear to contribute to tumor growth, metastasis, and recurrence in patients with colorectal cancer [75]. BCL9 has been shown to significantly enhance β‐catenin‐mediated transcription, thereby driving the expression of critical oncogenic targets, including c‐Myc and cyclin D1. This enhancement leads to an increase in tumor cell proliferation, migration, invasion, and metastatic potential, highlighting the critical role of BCL9 in cancer progression [76, 77, 78]. However, the role of BCL9 in BC has not been previously analyzed. In this study, we investigated the functional role of BCL9 in BC cells *in vitro* by conduction BCL9 knockdown experiments in the J82 and Cal29 cell lines. The knockdown of BCL9 led to a significant reduction in the proliferation, migration, and invasion of both cell lines without inducing apoptosis. Here, we demonstrate that BCL9 exhibits oncogenic properties in BC cells, which is consistent with its established role as a co‐activator within the Wnt/β‐catenin signaling pathway. This finding is consistent with the oncogenic function of BCL9 in other tumor entities. To the best of our knowledge, for the first time, we demonstrate that BCL9 and BCL9L, which are coactivators of Wnt/β‐catenin signaling, exert oncogenic effects in BC cells [38]. This assertion is further supported by the observation that the activation of the Wnt/β‐catenin signaling pathway, frequently induced by β‐catenin mutations, may contribute to the aggressive behavior of certain subtypes of bladder cancer [79].

Next, we focused on Wnt/β‐catenin inhibitors as potential therapeutic agents for BC. In our previous work, we demonstrated that the specific inhibitor iCRT3, which disrupts the protein interaction of β‐catenin with TCF/LEF transcription, inhibits the proliferation, migration and invasion of BC cells, thereby demonstrating the tumorigenic role of Wnt/β‐catenin in BC [38]. In the same context, the specific inhibitor ZW4864 was utilized, which binds to β‐catenin and disrupts the protein interaction of β‐catenin with the BCL9(L) protein, leading to the inhibition of Wnt/β‐catenin signaling [67]. In this study, we demonstrated by flexible CDOCKER analysis that ZW4864 effectively binds to the active site of β‐catenin, with CDOCKER energy value of −48.64 kcal/mol, indicating a strong binding affinity. These findings support the conclusion that ZW4864 occupies the BCL9 binding pocket on β‐catenin, thereby disrupting the β‐catenin/BCL9 protein–protein interaction, which is a critical driver of Wnt/β‐catenin signaling in cancer. Furthermore, protein–protein docking experiments using ZDOCK and RDOCK suggested that ZW4864 binds to the same active site in β‐catenin as BCL9 and BCL9L. This finding supports the hypothesis that ZW4864 has the potential to act as a competitive inhibitor. Overall, we conclude that ZW4864 demonstrate a binding affinity for the same active site of β‐catenin as BCL9. Furthermore, it has been observed that ZW4864 interact with key residues that are involved in BCL9 binding. Finally, the higher calculated binding affinity of ZW4864 with BCL9L exceeds that of BCL9.

In accordance with these findings, treatment of BC cells with ZW4864 resulted in a concentration‐dependent reduction in the expression of Wnt/β‐catenin target genes (AXIN2, LEF1, SP5, BIRC5, MMP9, and CCND1) in the BC cell lines Cal29 and J82, thereby suggesting that ZW4864 induces an inhibition of Wnt/β‐catenin signaling in BC. The observed concentration‐dependent inhibition in both cell lines supports the potential of the small‐molecule inhibitor ZW4864 as a pharmacologically relevant modulator of Wnt/β‐catenin signaling in BC models.

Therefore, we investigated whether ZW4864 has an antitumor effect in BC. The study demonstrated that the inhibition of Wnt/β‐catenin signaling by ZW4864 resulted in a dose‐dependent reduction in cell proliferation and induction of apoptosis at higher concentrations in the BC cell lines Cal29 and J82. Specifically, ZW4864 at concentration of 10, 20, and 40 μm demonstrated a significantly suppression of proliferation. At lower concentrations (10 μm and 20 μm), ZW4864 did not induce apoptosis, indicating a growth inhibitory effect at these concentrations. However, at a concentration of 40 μm, ZW4864 has been observed to induce significant increases in early apoptosis and cell death, accompanied by the upregulation of CDKN1A (p21), a cyclin‐dependent kinase inhibitor that is associated with cell cycle arrest and apoptosis [80]. Furthermore, 20 μm ZW4864 significantly inhibited the migration and invasion of Cal29 and J82 BC cells, as assessed by the xCELLigence real‐time cell analysis system. These findings are consistent with previous evidence that pharmacologic inhibition of Wnt/β‐catenin signaling, which is known to drive oncogenic behavior, inhibits tumorigenic properties in BC [38]. In summary, ZW4864 exerts both cytostatic and cytotoxic effects in a concentration‐dependent manner. This effect is achieved through the targeted inhibition of Wnt/β‐catenin/BCL9 signaling in BC cell lines.

The present study extends the characterization of the β‐catenin/BCL9 inhibitor ZW4864 from conventional 2D monolayer cultures to more physiologically relevant three‐dimensional (3D) bladder cancer models, including spheroids and multicellular BC organoids. The use of 3D culture systems has gained an increase recognition as essential component of preclinical drug evaluation. These models have been shown to more accurately recapitulate the tumor microenvironment, cellular heterogeneity, and drug resistance observed *in vivo* compared to 2D cultures [81]. Our findings demonstrate that ZW4864 effectively suppresses the growth of J82 BC spheroids in a dose‐dependent manner.

In comparison with conventional 2D monolayer models, multicellular BC organoid models offer significant advantages because they provide a more accurate representation of organ and tumor microenvironments. These advanced models are pivotal for understanding the oncogenic drivers and signaling pathways that contribute to tumor growth and cancer progression, making them essential tools in cancer research. Furthermore, organoids offer an alternative preclinical model for evaluating anticancer drugs and inhibitor, thereby eliminating the need for animal models and the ethical concerns that accompany them. This approach significantly accelerates the drug development process [82, 83, 84, 85]. For the cell‐based BC organoids, BC cell lines were mixed with human bladder fibroblasts (hBFs) and bladder smooth muscle cells (hBSMCs). Interestingly, these cells spontaneously form an organotypic self‐organized structure. hBF and hBSMC cells form a robust core, whereas BC cells simultaneously form an epithelial‐like layer at the periphery of BC organoids, resulting in the formation of distinct external and internal tissue‐like layers [59]. This model is particularly well suited for investigating the impact of BCL9L knockdown and ZW4864 on the self‐organization and structure of multicellular BC organoids. This is due to the fact that Wnt/β‐catenin signaling is involved in several associated biological processes, such as tissue integrity and homeostasis. Utilizing these models, we initiated to investigate the effects of BCL9L on tumor cell phenotype and the molecular mechanisms. Preliminary results indicate a potential impact on spheroid self‐assembly capacity and the expression of β‐catenin and EMT‐associated factors (data not shown). Subsequent studies are underway and will facilitate the elucidation of these mechanisms. Furthermore, we demonstrated that ZW4864 treatment during generation of BC organoids resulted in a significant decrease in the proliferation of BC cells and the thickness of the periphery layer of the organoids. Notably, the stromal core remained unaffected. The preservation of the stromal core under ZW4864 treatment indicates the specificity of this pathway in promoting tumor malignancy, as opposed to its general impairment of the multicellular organization of the organoids. The utilization of these cell‐based multicellular organoids holds particular relevance for the modeling tumor‐microenvironment interactions and the execution of drug screening procedures.

In summary, the antitumor effect of the commercially available inhibitor ZW4864 was demonstrated *in‐vitro* models of bladder cancer at a micromolar range. The development of this inhibitor was first published by Wang et al. in 2021 [86]. In the related study, Wang et al. treated mice harboring triple negative breast cancer PDX tumors with 90 mg/kg of the drug over 5 days, observing a slight but not significant reduction in tumor size. The study also noted that there was no significant decrease in body weight and no major toxicity issues were observed over the treatment period [67]. However, the clinical studies conducted on ZW4864 are limited and its clinical applicability remains to be elucidated. The administration of local, intravesical treatment for bladder cancer, either as a local adjuvant or a neoadjuvant in cases of local disease, presents a promising therapeutic strategy, provided that the toxicity to healthy tissue remains low. However, this hypothesis requires further investigation.

## 5 Conclusion

BCL9 and BCL9L have been identified as critical co‐activators of Wnt/β‐catenin signaling and play central roles in cancer progression. They promote the activation of oncogenic transcription programs, stemness, metastasis, and resistance to therapy. Our findings demonstrated that both BCL9 and BCL9L could function as oncogenes in bladder cancer, driving cell proliferation, migration and invasion. The targeting of β‐catenin/BCL9(L) axis has emerged as a promising strategy, particularly in the context of Wnt‐driven tumors. The small molecule inhibitor ZW4864 has been shown specifically to target the β‐catenin protein, thereby blocking its interaction with BCL9(L) proteins. This results in the repression of Wnt/β‐catenin signaling. In BC cell lines, ZW4864 has been shown to downregulate the expression of Wnt/β‐catenin target genes, and suppress tumor cell growth, migration, and invasion in two‐dimensional models of BC cells. Interestingly, the use of multicellular bladder cancer organoid models has been shown to more accurately recapitulate the tumor microenvironment and heterogeneity. These models have also demonstrated the critical role of Wnt/β‐catenin/BCL9(L) signaling in the proliferation and maintenance of malignant phenotypes within three‐dimensional culture systems. These findings highlight the therapeutic potential of targeting the β‐catenin/BCL9(L) axis in bladder cancer and support the use of advanced organoid models for preclinical evaluation of Wnt/β‐catenin pathway inhibitors.

## Conflict of interest

The authors have no relevant financial or nonfinancial interests to disclose.

## Author contributions

DS and MOG conceptualized and supervised the project; DS and RK designed the methodology; RK carried out the experiments and formal analysis; EL carried out the qPCR experiments; MBP performed the experiments and analysis of the organoids; GN, AA, and CG designed, performed, and analyzed the experiments for the porcine urinary organ model; KPS, SKG, and OW performed the docking experiments; RK and DS wrote the manuscript; OH advised the project and co‐wrote the manuscript. All the authors have read and agreed with the published version of the manuscript.

## Supporting information


**Fig. S1.** Whole PVDF membrane of the western blot to estimate the protein expression of BCL9L in Cal29 cells after stable knockdown of BCL9L experiments (Clone a, d, e) at different time points of cultivation, Membrane A and B. The whole membrane was cut horizontal and the two parts were detected simultaneously for BCL9L (upper part) and GAPDH (lower part), respectively. Molecular weight marker: ThermoScientific PageRuler Plus.
**Fig. S2.** Whole PVDF membrane of the western blot to estimate the protein expression of BCL9 after BCL9 knockdown in TCCsup, Cal29 and J82 cells. The whole membrane was cut horizontal and the two parts were detected simultaneously for BCL9 (upper part) and GAPDH (lower part). Molecular weight marker: ThermoScientific PageRuler Plus.
**Fig. S3.** (A) Control experiment for apoptosis. The Cal29 and J82 cells were treated with 10 μm Camptothecin and were analyzed for apoptosis after 24 h. The apoptosis assay was performed by dual staining with annexin V‐FITC and propidium iodide (PI) kit and analyzed by flow cytometry. The data are expressed as mean ± standard deviation of three experiments and statistical analysis was performed by two‐tailed unpaired Student's *t*‐test with *P ≤ 0.05, **P ≤ 0.01, ***P ≤ 0.001. *n*: independent biological replicates. (B) Apoptosis analyzes by flow cytometry using dual staining with an annexin V‐FITC and propidium iodide (PI) kit of Cal29 and J82 cells after treatment with 10 μm, 20 μm or 40 μm ZW4864 and analyzed after 2 days.
**Fig. S4.** All three independent biological replicates migration and invasion experiments analyzed by xCelligence system after BCL9 knockdown. For the migration, 20.000 pre‐transfected Cal29 and J82 cells with siBCL9 or siControl were seeded into CIM plate and analyzed. For invasion, 40.000 pretransfected Cal29 and J82 cells were seeded into CIM plate precoated with matrigel and analyzed. The migration and invasion are reduced in Cal29 and J82 cells after knockdown of BCL9. The data are expressed as mean ± standard deviation of three technical replicates. The cell index values correspond to the cell number of the migrated or invasive cells.
**Fig. S5.** Real‐time migration and invasion experiments of Cal29 and J82 after BCL9/β‐catenin inhibitor ZW4864 treatment. All three independent biological replicates are shown. For the migration, 20.000 pretreated cells with 20 μM ZW4864 were seeded into CIM plate and analyzed by xCELLigence system. For invasion, 40.000 pretreated cells with 20 μM ZW4864 were seeded into CIM plate precoated with matrigel and analyzed. The data are expressed as mean ± standard deviation of three technical replicates. The cell index values correspond to the cell number that migrates or invades.
**Fig. S6.** Live cell imaging of cytotoxic effects of the inhibitor ZW4864 in pre‐formed (multilayered) CAL29 and J82 organoids. The inhibitor ZW4864 (10, 20, 40 μm) was added after 3 days of CAL29 (A) and J82 (B) organoid culture; plates were placed into the Incucyte Live‐Cell Analysis System (Sartorius, Epsom, UK). Repeated scanning every 4 h over a period of 10 days was scheduled for live cell imaging. ZW4864 treatment resulted in dissociation of BC organoids especially at 40 μm ZW4864. Scale bar: 800 μm.
**Fig. S7.** Tumor‐selective effects of the BCL9/β‐catenin inhibitor ZW4864 in pre‐formed (multilayered) J82 organoids. The inhibitor ZW4864 (20 μm) was added after 3 days of organoid culture; organoids were fixed after 3, 7 and 10 days of organoid culture. (A) Representative images of J82 organoids (ZW4864, vs. DMSO) immunostained for panCK, vimentin, αSMCA and KI67 after 10 days of ZW4864 treatment. Scale bar: 150 μm. (B) Quantification of organoid size, BC layer thickness and diameter of stroma cell core. BCL9/β‐catenin inhibition resulted in significantly decreased BC layer after 10 days, while the stroma cell core was not affected; * significant differences compared to vehicle control (DMSO); *P* ≤ 0.05, Mann–Whitney test, mean + SD.
**Table S1.** Details of the atomic interactions and bond parameters for β‐catenin complexes with BCL9, BCL9L, and ZW4864. The table lists the atoms involved in bond formation, bond lengths (in ångströms, Å), bond types, and corresponding interaction energies. The interactions between β‐catenin and BCL9/BCL9L were identified through protein–protein docking, whereas the β‐catenin–ZW4864 interactions were obtained using protein–ligand docking.
**Table S2.** Top 10 unique human protein targets of ZW4864, ranked by decreasing Fit‐Value using the *Ligand Profiler* protocol in DS2022, with corresponding PDB IDs and full protein names.
**Table S3.** Enrichment analysis of the top 10 off‐targets for ZW4864, with targets ranked according to their fold enrichment score.

## Data Availability

The data that supports the findings of this study are available in this article and the supplementary material. Additional data are available from the corresponding author upon reasonable request.
