# Supplementary material for: ZW4864‐mediated inhibition of the β‐catenin/BCL9/BCL9L complex reveals therapeutic potential in bladder cancer
Source: Mol Oncol. 2026 Jun 18:10.1002/1878-0261.70292. Online ahead of print. doi: 10.1002/1878-0261.70292 (PMC13398921; doi:10.1002/1878-0261.70292)
Supplement: Supplementary file 1 — Fig. S1. Whole PVDF membrane of the western blot to estimate the protein expression of BCL9L in Cal29 cells after stable knockdown of BCL9L experiments (Clone a, d, e) at different time points of cultivation, Membrane A and B. The whole membrane was cut horizontal and the two parts were detected simultaneously for BCL9L (upper part) and GAPDH (lower part), respectively. Molecular weight marker: ThermoScientific PageRuler Plus. Fig. S2. Whole PVDF membrane of the western blot to estimate the protein expression of BCL9 after BCL9 knockdown in TCCsup, Cal29 and J82 cells. The whole membrane was cut horizontal and the two parts were detected simultaneously for BCL9 (upper part) and GAPDH (lower part). Molecular weight marker: ThermoScientific PageRuler Plus. Fig. S3. (A) Control experiment for apoptosis. The Cal29 and J82 cells were treated with 10 μm Camptothecin and were analyzed for apoptosis after 24 h. The apoptosis assay was performed by dual staining with annexin V‐FITC and propidium iodide (PI) kit and analyzed by flow cytometry. The data are expressed as mean ± standard deviation of three experiments and statistical analysis was performed by two‐tailed unpaired Student's t‐test with *P ≤ 0.05, **P ≤ 0.01, ***P ≤ 0.001. n: independent biological replicates. (B) Apoptosis analyzes by flow cytometry using dual staining with an annexin V‐FITC and propidium iodide (PI) kit of Cal29 and J82 cells after treatment with 10 μm, 20 μm or 40 μm ZW4864 and analyzed after 2 days. Fig. S4. All three independent biological replicates migration and invasion experiments analyzed by xCelligence system after BCL9 knockdown. For the migration, 20.000 pre‐transfected Cal29 and J82 cells with siBCL9 or siControl were seeded into CIM plate and analyzed. For invasion, 40.000 pretransfected Cal29 and J82 cells were seeded into CIM plate precoated with matrigel and analyzed. The migration and invasion are reduced in Cal29 and J82 cells after knockdown of BCL9. The data are expressed as m [file MOL2-9999-0-s001.docx]

**Supplementary**

**ZW4864-mediated inhibition of the β-catenin/BCL9/BCL9L complex reveals therapeutic potential in bladder cancer**

Roland Kotolloshi^1, 2, 7^, Mandy Berndt-Paetz^3, 7^, Eileen Lerner^1^, Gregoire Najjar^4^, Anca Azoitei^4^, Krishna Pal Singh^5, 6^, Shailendra Kumar Gupta^5^, Olaf Wolkenhauer^5^, Cagatay Günes^4, 9^, Otmar Huber^7^, Marc-Oliver Grimm^1^ and Daniel Steinbach^1, 8, 9 *^

^1^ Department of Urology, Jena University Hospital, 07747 Jena, Germany

^2^ Department of Urology, University Medical Center Rostock, 18057 Rostock, Germany.

^3^ Department of Urology, Leipzig University, 04103 Leipzig, Germany

^4^ Department of Urology, Ulm University Hospital, 89081 Ulm, Germany

^5^ Department of Systems Biology & Bioinformatics, Institute of Computer Science, University of Rostock, 18059 Rostock, Germany

^6^ Clinic and Policlinic for Dermatology, Venereology and Allergology, University Medical Center Rostock, 18059 Rostock, Germany

^7^ Department of Biochemistry II, Jena University Hospital, Jena, Germany

^8^ UroFors Consortium (Natural Scientists in Urological Research), German Society of Urology, Düsseldorf, Germany.

^9^ German Study Group of Bladder Cancer (DFBK e.V.), Munich, Germany.

^*^Corresponding author:

Email: [d.steinbach@med.uni-jena.de](mailto:d.steinbach@med.uni-jena.de); Tel.: +49 3641 9390880; ORCID 0000-0001-5025-7347


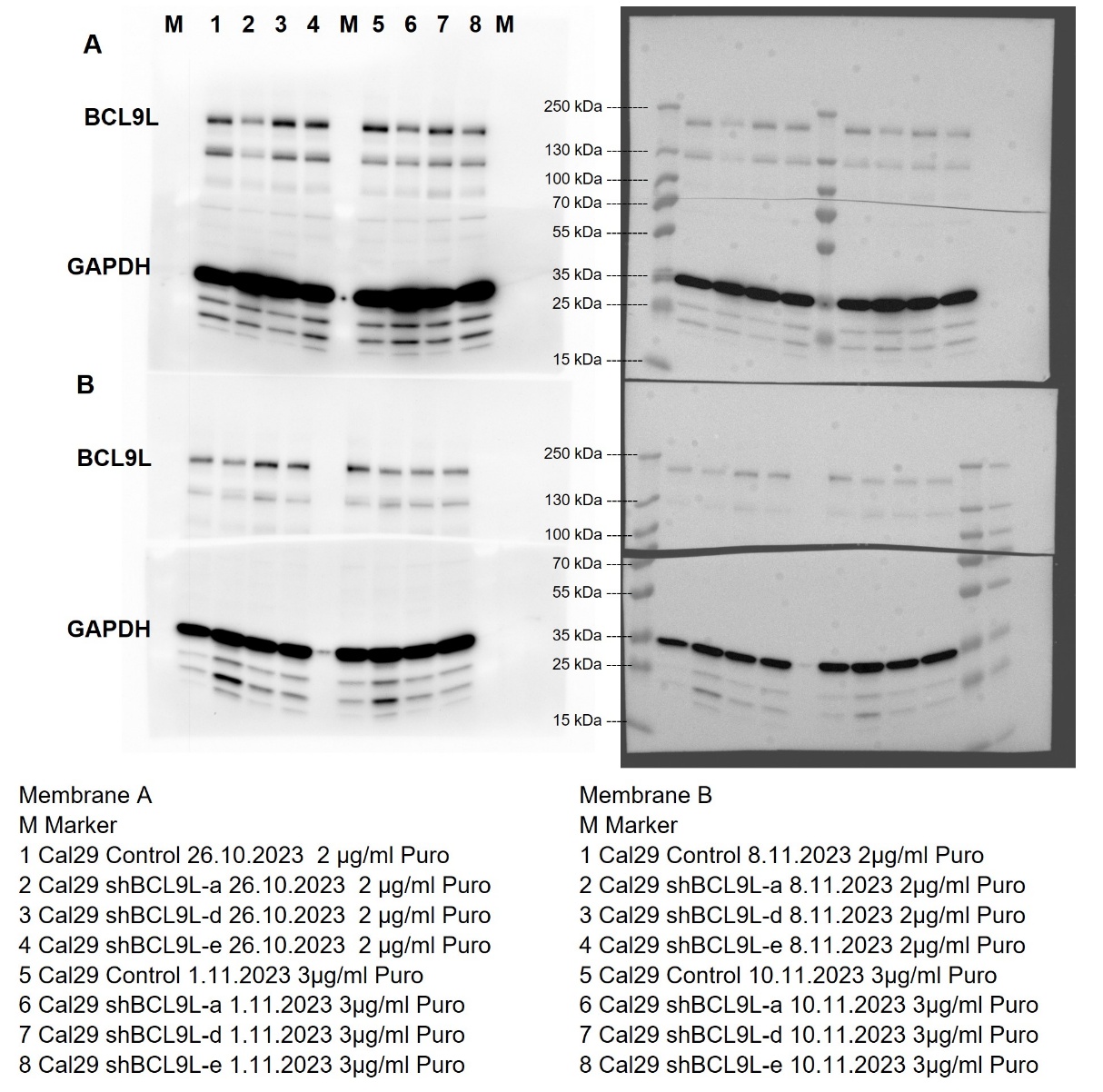
**Supplementary Figure S1.** Whole PVDF membrane of the Western blot to estimate the protein expression of BCL9L in Cal29 cells after stable knockdown of BCL9L experiments (Clone a, d, e) at different time points of cultivation, Membrane A and B. The whole membrane was cut horizontal and the two parts were detected simultaneously for BCL9L (upper part) and GAPDH (lower part), respectively. Molecular weight marker: ThermoScientific PageRuler Plus.


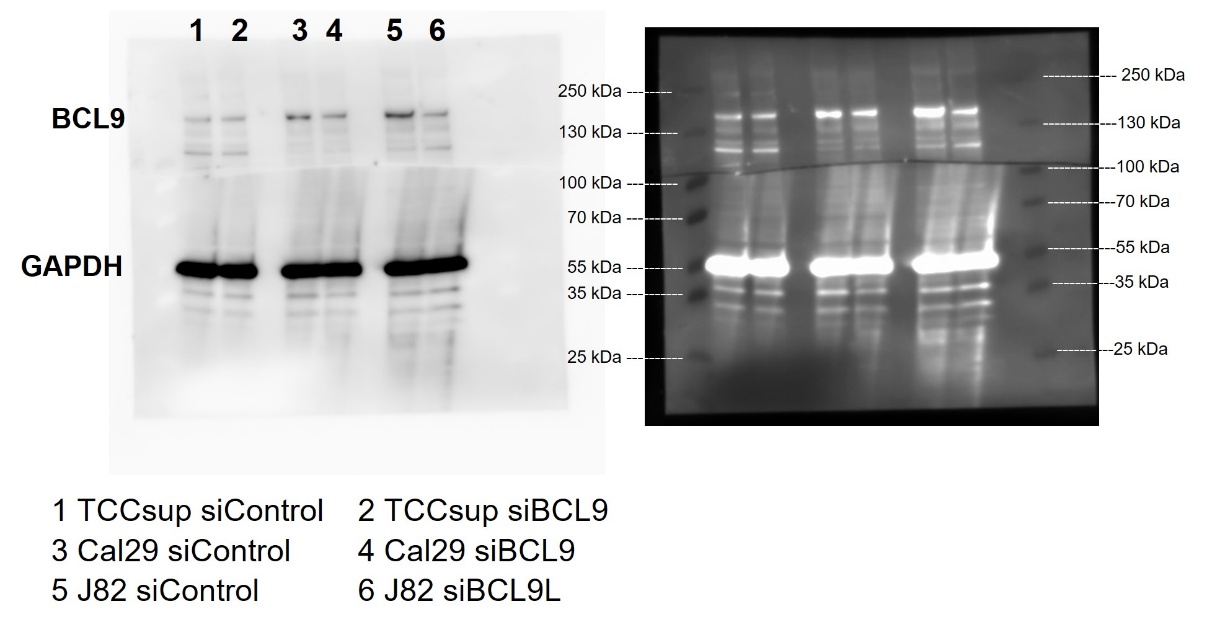


**Supplementary Figure S2.** Whole PVDF membrane of the Western blot to estimate the protein expression of BCL9 after BCL9 knockdown in TCCsup, Cal29 and J82 cells. The whole membrane was cut horizontal and the two parts were detected simultaneously for BCL9 (upper part) and GAPDH (lower part). Molecular weight marker: ThermoScientific PageRuler Plus.


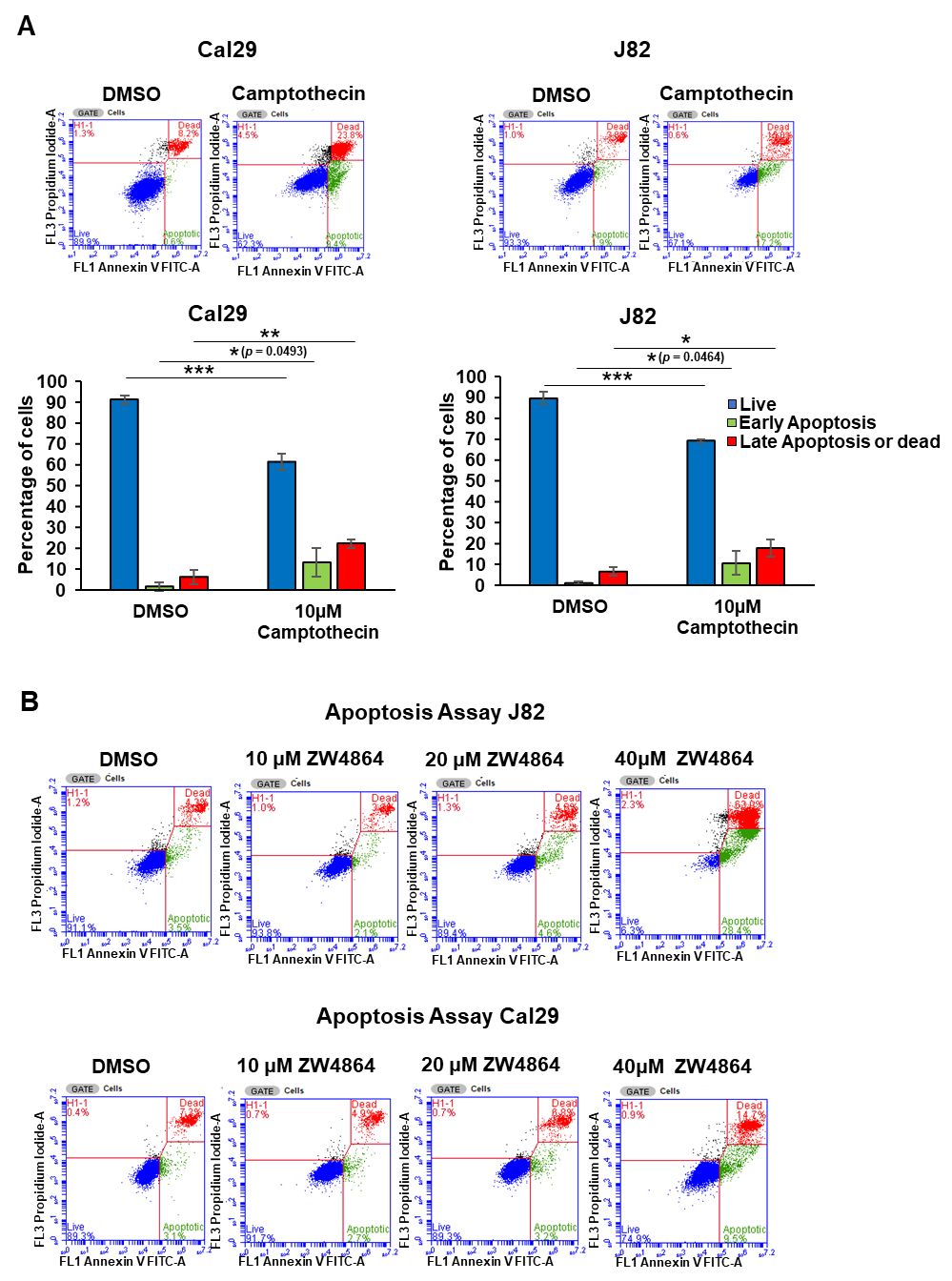


**Supplementary Figure S3.**

(A) Control experiment for apoptosis. The Cal29 and J82 cells were treated with 10 μM Camptothecin and were analysed for apoptosis after 24 hr. The apoptosis assay was performed by dual staining with annexin V-FITC and propidium iodide (PI) kit and analysed by flow cytometry. The data are expressed as mean ± standard deviation of three experiments and statistical analysis was performed by two-tailed unpaired Student’s t-test with * p ≤ 0.05, ** p ≤ 0.01, *** p ≤ 0.001. n: independent biological replicates.

(B) Apoptosis analyzes by flow cytometry using dual staining with an annexin V-FITC and propidium iodide (PI) kit of Cal29 and J82 cells after treatment with 10 μM, 20 μM or 40 μM ZW4864 and analyzed after 2 days.


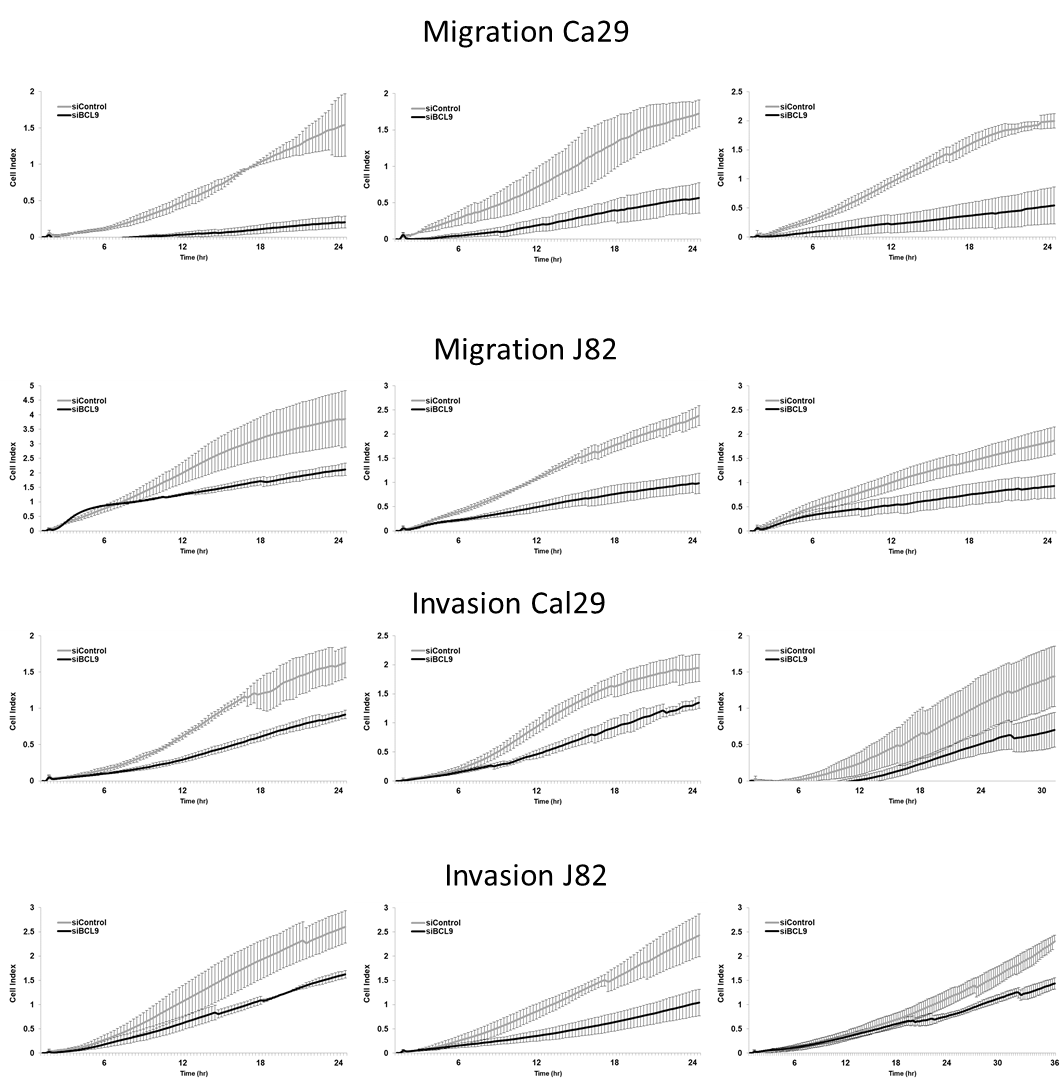


**Supplementary Figure S4.** All three independent biological replicates migration and invasion experiments analysed by xCelligence system after BCL9 knockdown. For the migration, 20.000 pre-transfected Cal29 and J82 cells with siBCL9 or siControl were seeded into CIM plate and analysed. For invasion, 40.000 pre-transfected Cal29 and J82 cells were seeded into CIM plate pre-coated with matrigel and analysed. The migration and invasion are reduced in Cal29 and J82 cells after knockdown of BCL9. The data are expressed as mean ± standard deviation of three technical replicates. The cell index values correspond to the cell number of the migrated or invasive cells.

**
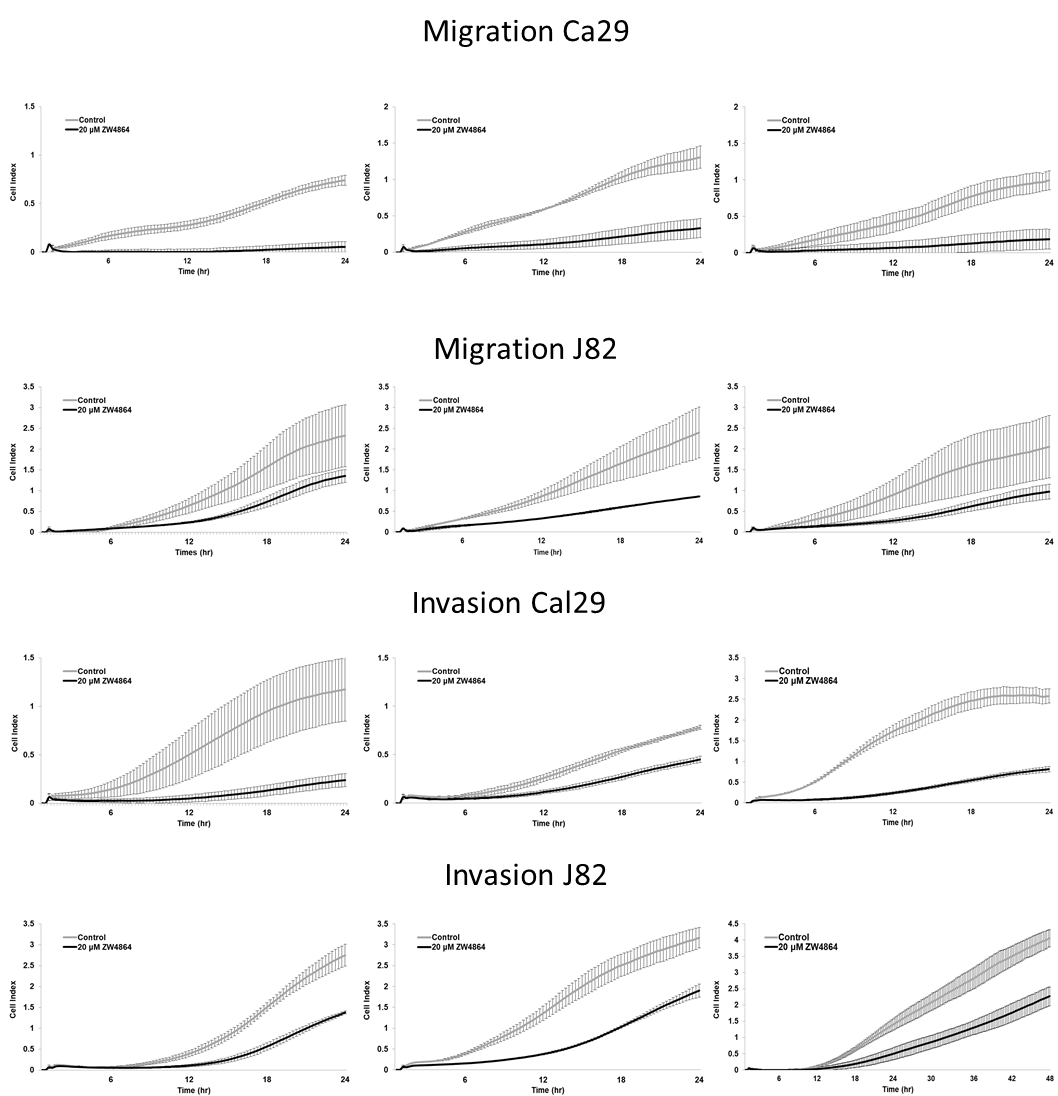
**

**Supplementary Figure S5.** Real-time migration and invasion experiments of Cal29 and J82 after BCL9/β-catenin inhibitor ZW4864 treatment. All three independent biological replicates are shown. For the migration, 20.000 pre-treated cells with 20 μM ZW4864 were seeded into CIM plate and analysed by xCELLigence system. For invasion, 40.000 pre-treated cells with 20 μM ZW4864 were seeded into CIM plate pre-coated with matrigel and analysed. The data are expressed as mean ± standard deviation of three technical replicates. The cell index values correspond to the cell number that migrates or invades.

**
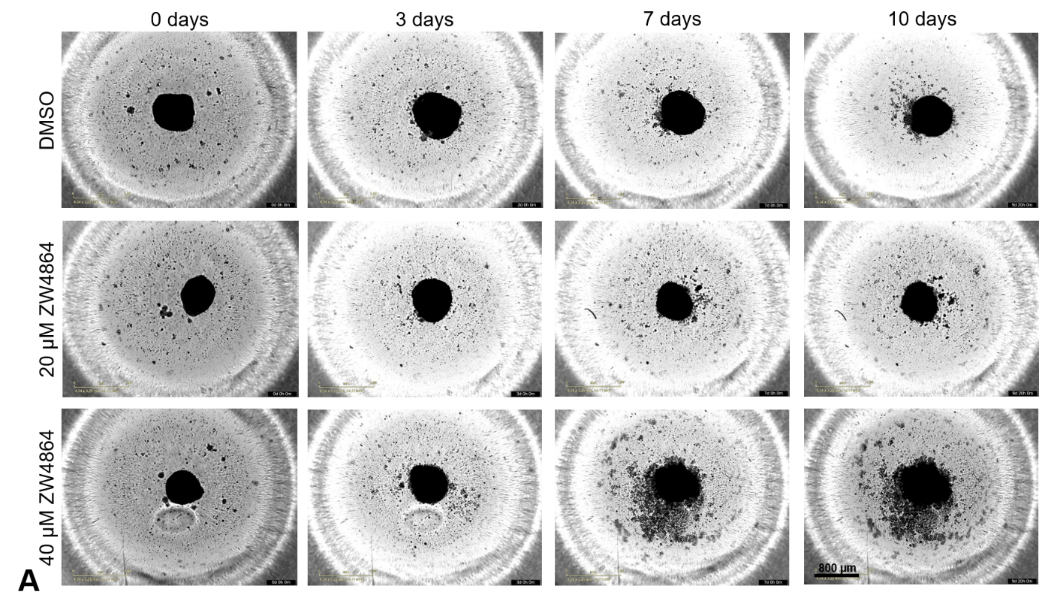
**

**
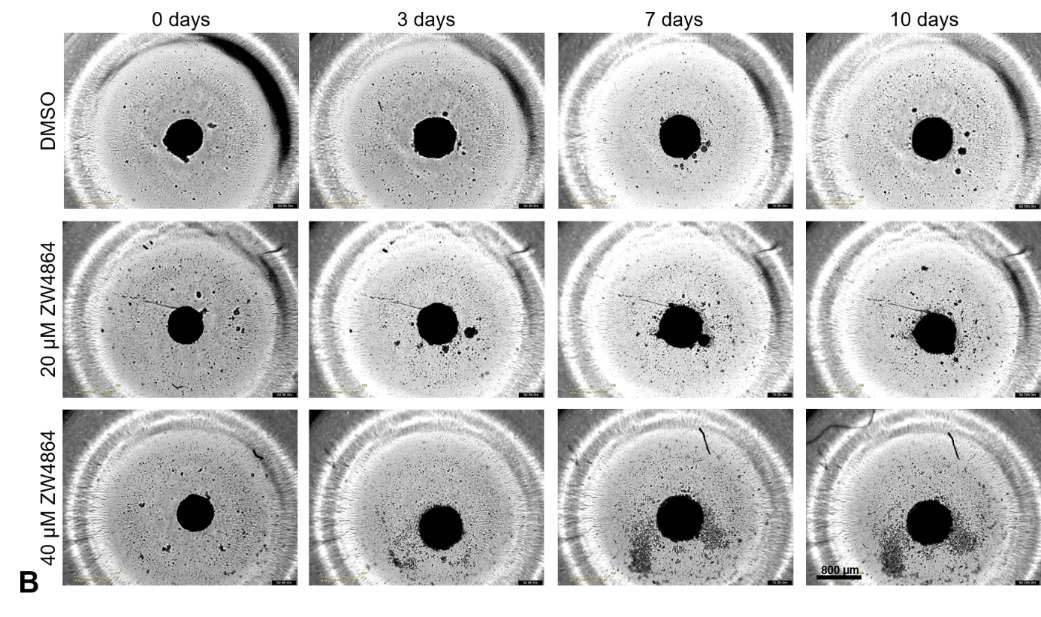
**

**Supplementary Figure S6****.** Live cell imaging of cytotoxic effects of the inhibitor ZW4864 in pre-formed (multilayered) CAL29 and J82 organoids. The inhibitor ZW4864 (10, 20, 40 µM) was added after 3 days of CAL29 (A) and J82 (B) organoid culture; plates were placed into the Incucyte^®^ Live-Cell Analysis System (Sartorius, Epsom, UK). Repeated scanning every 4 h over a period of 10 days was scheduled for live cell imaging. ZW4864 treatment resulted in dissociation of BC organoids especially at 40 μM ZW4864. Scale bar: 800 µm.


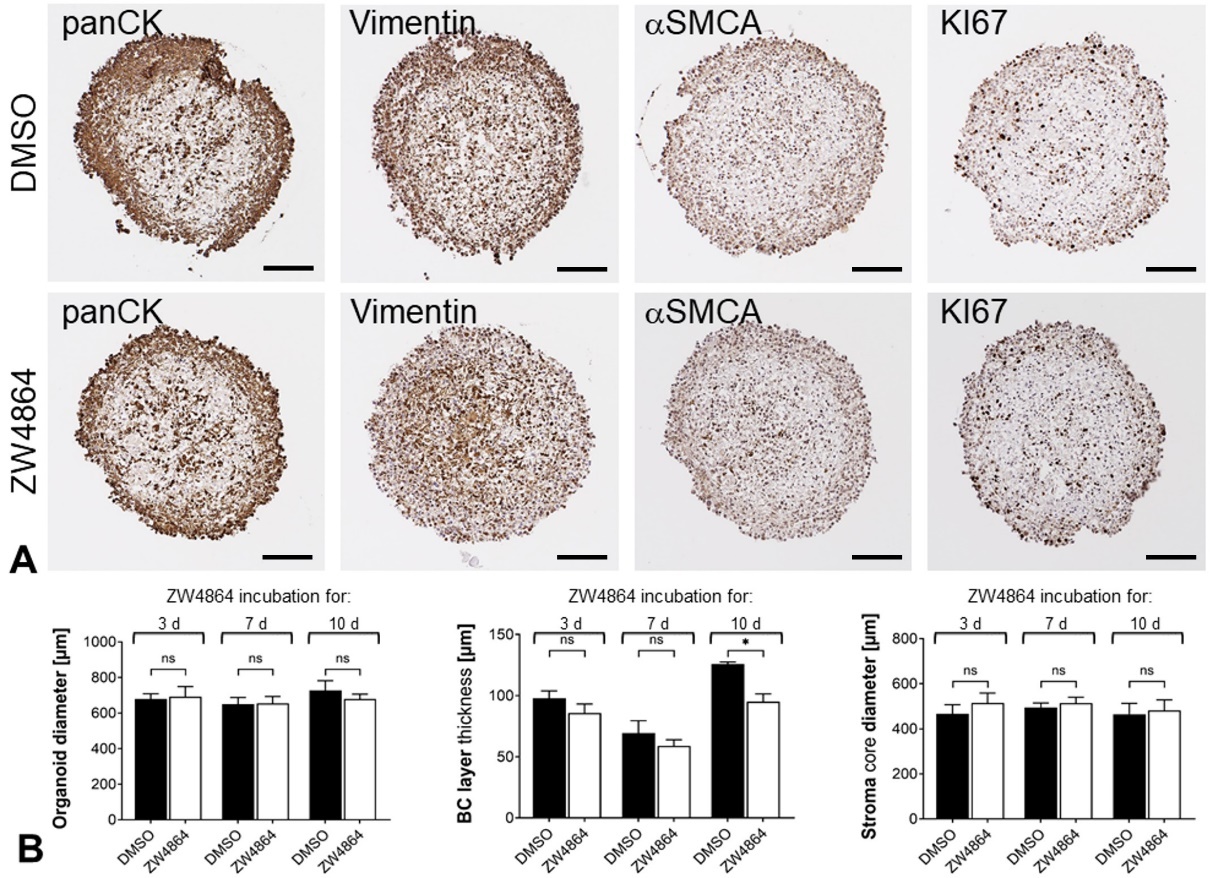


**Supplementary Figure S7.** Tumor-selective effects of the BCL9/β-catenin inhibitor ZW4864 in pre-formed (multilayered) J82 organoids. The inhibitor ZW4864 (20 µM) was added after 3 days of organoid culture; organoids were fixed after 3, 7 and 10 days of organoid culture. (A) Representative images of J82 organoids (ZW4864, vs. DMSO) immunostained for panCK, vimentin, αSMCA and KI67 after 10 days of ZW4864 treatment. Scale bar: 150 µm. (B) Quantification of organoid size, BC layer thickness and diameter of stroma cell core. BCL9/β-catenin inhibition resulted in significantly decreased BC layer after 10 days, while the stroma cell core was not affected; * significant differences compared to vehicle control (DMSO); p ≤ 0.05, Mann-Whitney test, mean + SD.

**Supplementary Table S1**

Details of the atomic interactions and bond parameters for β-catenin complexes with BCL9, BCL9L, and ZW4864. The table lists the atoms involved in bond formation, bond lengths (in ångströms, Å), bond types, and corresponding interaction energies. The interactions between β-catenin and BCL9/BCL9L were identified through protein–protein docking, whereas the β-catenin–ZW4864 interactions were obtained using protein–ligand docking.

| **β-catenin / BCL9L** | **Bond length (Å)** | **Bond category** | **E_RDOCK Energy (kcal/mol)** | **Protocol used** |
| --- | --- | --- | --- | --- |
| β-catenin:ARG151:NH1 - BCL9L:ASP256:OD2 | 4.42547 | Electrostatic | -10.5069 | **Protein- protein docking**  **(ZDOCK, RDOCK)** |
| β-catenin:LYS170:HZ1 - BCL9L:PHE238:O | 2.16982 | Hydrogen Bond |  |  |
| β-catenin:LYS170:HZ3 - BCL9L:PHE238:O | 2.09266 | Hydrogen Bond |  |  |
| β-catenin:LYS181:HZ1 - BCL9L:LEU251:O | 2.17214 | Hydrogen Bond |  |  |
| β-catenin:ARG151:CD - BCL9L:ASP256:O | 3.13599 | Hydrogen Bond |  |  |
| BCL9L:GLY253:CA -  β-catenin:GLU147:OE1 | 3.35189 | Hydrogen Bond |  |  |
| β-catenin:LEU148 - BCL9L:LEU251 | 5.41716 | Hydrophobic |  |  |
| β-catenin:ALA152 - BCL9L:VAL250 | 4.02116 | Hydrophobic |  |  |
| β-catenin:LEU156 - BCL9L:ILE258 | 4.85559 | Hydrophobic |  |  |
| β-catenin:MET174 - BCL9L:ILE258 | 4.69601 | Hydrophobic |  |  |
| β-catenin:LEU178 - BCL9L:LEU251 | 4.45985 | Hydrophobic |  |  |
| β-catenin:LYS181 - BCL9L:LEU251 | 4.58932 | Hydrophobic |  |  |
| BCL9L:ALA243 -  β-catenin:MET174 | 3.87333 | Hydrophobic |  |  |
| BCL9L:ALA246 -  β-catenin:MET174 | 5.27708 | Hydrophobic |  |  |
| BCL9L:ALA247 -  β-catenin:MET174 | 4.21195 | Hydrophobic |  |  |
| BCL9L:ALA247 -  β-catenin:LEU178 | 4.38077 | Hydrophobic |  |  |
| **β-catenin / BCL9** | **Bond length (Å)** | **Bond category** | **E_RDOCK Energy (kcal/mol)** |  |
| BCL9:ARG359:HH12 -  β-catenin:ASP164:OD2 | 2.70921 | Hydrogen Bond; Electrostatic | -22.4073 |  |
| BCL9:ARG359:NH1 -  β-catenin:ASP162:OD2 | 3.53234 | Electrostatic |  |  |
| β-catenin:LYS181:HZ2 - BCL9:LEU373:O | 2.09814 | Hydrogen Bond |  |  |
| BCL9:ARG359:HH21 -  β-catenin:ASP162:OD1 | 2.39849 | Hydrogen Bond |  |  |
| β-catenin:LEU148 -  BCL9:LEU373 | 4.91598 | Hydrophobic |  |  |
| β-catenin:ALA152 -  BCL9:LEU366 | 5.16885 | Hydrophobic |  |  |
| β-catenin:ALA152 -  BCL9:ILE369 | 3.87845 | Hydrophobic |  |  |
| β-catenin:LEU156 - BCL9:LEU366 | 4.08548 | Hydrophobic |  |  |
| β-catenin:LEU159 - BCL9:LEU363 | 4.76835 | Hydrophobic |  |  |
| β-catenin:MET174 - BCL9:LEU366 | 5.06326 | Hydrophobic |  |  |
| BCL9:ARG359 -  β-catenin:VAL167 | 5.43864 | Hydrophobic |  |  |
| BCL9:PHE374 -  β-catenin:LYS181 | 4.5356 | Hydrophobic |  |  |
| **β-catenin / ZW4864** | **Bond length (Å)** | **Bond category** | **CDOCKEREnergy (kcal/mol)** |  |
| β-catenin:GLN177:HE21 - ZW4864:O1 | 2.89755 | Hydrogen Bond | -48.64 | **Protein-ligand docking (DCOCKER)** |
| β-catenin:HIS176:HD2 - ZW4864:O3 | 2.34902 | Hydrogen Bond |  |  |
| ZW4864:H71 -  β-catenin:GLU209:OE2 | 2.54304 | Hydrogen Bond |  |  |
| ZW4864:H84 -  β-catenin:LYS170:O | 2.48892 | Hydrogen Bond |  |  |
| ZW4864 -  β-catenin:MET174 | 5.42424 | Hydrophobic |  |  |
| β-catenin :HIS176 -  ZW4864 | 5.35939 | Hydrophobic |  |  |
| ZW4864 -  β-catenin:LEU159 | 5.35501 | Hydrophobic |  |  |
| ZW4864 -  β-catenin:ALA171 | 5.20732 | Hydrophobic |  |  |
| ZW4864 -  β-catenin:VAL173 | 4.56386 | Hydrophobic |  |  |

**Supplementary Table S2.** Top ten unique human protein targets of ZW4864, ranked by decreasing Fit-Value using the *Ligand Profiler* protocol in DS2022, with corresponding PDB IDs and full protein names.

| **Rank** | **PDB ID** | **Target Full Name** | **Fit-Value** |
| --- | --- | --- | --- |
| 1 | 1OKN | Carbonic anhydrase 2 (CA2) | 0.815019 |
| 2 | 2HRC | Ferrochelatase, mitochondrial (FECH) | 0.79691 |
| 3 | 2NMX | Carbonic anhydrase 1 (CA1) | 0.740541 |
| 4 | 3EKR | Heat shock protein HSP 90-alpha (HSP90AA1) | 0.720159 |
| 5 | 3R28 | Cyclin-dependent kinase 2 (CDK2) | 0.675595 |
| 6 | 4A9L | Bromodomain-containing protein 4 (BRD4) | 0.635957 |
| 7 | 3AC1 | Tyrosine-protein kinase Lck (LCK) | 0.626841 |
| 8 | 3BYZ | 11-beta-hydroxysteroid dehydrogenase 1 (HSD11B1) | 0.604339 |
| 9 | 2V5Z | Amine oxidase [flavin-containing] B (MAOB) | 0.603086 |
| 10 | 3X03 | Phosphatidylinositol 5-phosphate 4-kinase type-2 beta (PIP4K2B) | 0.602913 |

**Supplementary Table S3**

Enrichment analysis of the top 10 off-targets for ZW4864, with targets ranked according to their fold enrichment score.

| Enrichment FDR | nGenes | Pathway Genes | Fold Enrichment | Pathway (KEGG) | Genes |
| --- | --- | --- | --- | --- | --- |
| 0.000739383 | 2 | 17 | 268.188 | Nitrogen metabolism | CA2; CA1 |
| 0.012300359 | 2 | 97 | 47.002 | Prostate cancer | HSP90AA; CDK2 |
| 0.012300359 | 2 | 100 | 45.592 | Progesterone-mediated oocyte maturation | HSP90AA1; CDK2 |
| 0.012300359 | 2 | 108 | 42.2148 | Th17 cell differentiation | HSP90AA1; LCK |
| 0.042372335 | 2 | 222 | 20.536 | Human T-cell leukemia virus 1 infection | CDK2; LCK |
| 0.000739383 | 6 | 1527 | 8.9571 | Metabolic pathways | FECH; MAOB; CA2; HSD11B1; CA1; PIP4K2B |
